# Inhibition of growth and contraction in human prostate stromal cells by silencing of NUAK1 and -2, and by the presumed NUAK inhibitors HTH01-015 and WZ4003

**DOI:** 10.3389/fphar.2023.1105427

**Published:** 2023-04-28

**Authors:** Yuhan Liu, Ruixiao Wang, Ru Huang, Beata Rutz, Anna Ciotkowska, Alexander Tamalunas, Sheng Hu, Moritz Trieb, Raphaela Waidelich, Frank Strittmatter, Christian G. Stief, Martin Hennenberg

**Affiliations:** Department of Urology, University Hospital Munich, LMU Munich, Munich, Germany

**Keywords:** benign prostatic hyperplasia (BPH), lower urinary tract symptoms (LUTS), voiding symptoms, bladder outlet obstruction (BOO), prostate smooth muscle contraction, prostate growth, prostate enlargement

## Abstract

**Background:** NUAKs promote myosin light chain phosphorlyation, actin organization, proliferation and suppression of cell death in non-muscle cells, which are critical for smooth muscle contraction and growth. In benign prostatic hyperplasia (BPH), contraction and growth in the prostate drive urethral obstruction and voiding symptoms. However, a role of NUAKs in smooth muscle contraction or prostate functions are unknown. Here, we examined effects of NUAK silencing and the presumed NUAK inhibitors, HTH01-015 and WZ4003 on contraction and growth-related functions in prostate stromal cells (WPMY-1) and in human prostate tissues.

**Methods:** Effects of NUAK1 and -2 silencing, HTH01-015 and WZ4003 on matrix plug contraction, proliferation (EdU assay, Ki-67 mRNA), apoptosis and cell death (flowcytometry), viability (CCK-8) and actin organization (phalloidin staining) were examined in cultured WPMY-1 cells. Effects of HTH01-015 and WZ4003 on smooth muscle contraction were assessed in organ bath experirments with human prostate tissues.

**Results:** Effects of silencing were most pronounced on proliferation and cell death, resulting in decreases of proliferation rate by 60% and 70% by silencing of NUAK1 and NUAK2 (compared to scramble siRNA-transfected controls), decreases in Ki-67 by 75% and 77%, while numbers of dead cells after silencing of NUAK1 and NUAK2 amounted to 2.8 and 4.9 fold of scramble-transfected controls. Silencing of each isoform was paralleled by reduced viability, breakdown in actin polymerization, and partial decreases in contractility (maximally 45% by NUAK1 silencing, 58% by NUAK2 silencing). Effects of silencing were mimicked by HTH01-015 and WZ4003, with numbers of dead cells amounting up to 16.1 fold or 7.8 fold with HTH01-015 or WZ4003, compared to solvent-treated controls. Using concentrations of 500 nM, neurogenic contractions of prostate tissues were inhibited partly by HTH01-015 and U46619-induced contractions were inhibited partly by HTH01-015 and WZ4003, while α_1_-adrenergic and endothelin-1-induced contractions remained unaffected. Using 10 μM, inhibition of endothelin-1-induced contractions by both inhibitors and inhibition of α_1_-adrenergic contractions by HTH01-015 added to effects seen by 500 nM.

**Conclusion:** NUAK1 and -2 suppress cell death and promote proliferation in prostate stromal cells. A role in stromal hyperplasia appears possible in BPH. Effects of NUAK silencing are mimicked by HTH01-015 and WZ4003.

## 1 Background

Voiding and post-void symptoms suggestive of benign prostatic hyperplasia (BPH) are characterized by high prevalence, while their medical treatment still remains challenging. Around 50% of men in the sixth life decade and 90% in the ninth decade show histologically proven BPH, while 35% of men in the eight life decade experience micturition problems (Lepor, 2004). Due to the age-dependency of prevalence together with the demographic transition, case numbers are continuously increasing (Irwin et al., 2011). Estimations for the year 2018 amounted the worldwide number of male patients with voiding symptoms to 612 million, and to 332 million cases of post-void symptoms (Irwin et al., 2011). Driving factors of voiding symptoms suggestive BPH are an increased prostate smooth muscle tone and benign prostatic growth (Lepor, 2004). Both factors may contribute to urethral compression, finally resulting in impairments of urinary flow and bladder emptying (Lepor, 2004). Consequently, medical treatment is based on α_1_-adrenoceptor antagonists (α_1_-blockers) and the phosphodiesterase-5 inhibitor tadalafil to inhibit prostate smooth muscle contraction, and 5α-reductase inhibitors (5-ARI) to reduce prostate growth (Oelke et al., 2013). However, α_1_-blockers improve symptoms and urinary flow by no more than 50%, and 5-ARI reduce prostate size only up to 28% (Oelke et al., 2013). Combination therapies are commonly applied, but often discontinued due to low tolerability, resulting in progression, complications and surgery for BPH (Oelke et al., 2013; Cindolo et al., 2015a; Cindolo et al., 2015b).

In face of these limitations, together with increasing case numbers, novel medications are of high demand, which requires adequate understanding of molecular mechanisms underlying prostate smooth muscle contraction and growth. Previous basic and clinical research mainly focused on α_1_-adrenoceptors and three intracellular signaling pathways (calcium, protein kinase C, RhoA/Rho kinase) in the context of prostate smooth muscle contraction (Hennenberg et al., 2014). More recent findings suggested, that limitations of α_1_-blockers may be caused by non-adrenergic contractions of prostate smooth muscle, and that intracellular signaling in prostate smooth contraction is much more complex than previously assumed (Hennenberg et al., 2014). Generally, novel targets and compounds with impacts on actin organization or myosin light chain (MLC) phosphorylation may be considered in the context of BPH, as actin organization is an essential prerequisite for smooth muscle contraction and proliferation, and MLC phosphorylation is required for smooth muscle contraction (Hennenberg et al., 2014).

NUAK1 and -2 are two members of the AMP-activated protein kinase (AMPK) family (Faisal et al., 2021). A role for regulation of the MLC-targeting MLC phosphatase subunit MYPT1 and, accordingly, for MLC phosphorylation has been demonstrated (Sun et al., 2013; Faisal et al., 2021), but a role of NUAKs for smooth muscle contraction has not been examined to date. Both isoforms may promote MLC phosphorylation, being preceded by phosphorylation of MYPT1 (Faisal et al., 2021). NUAK-mediated phosphorylation of MYPT1 occurs at serine-445, -472 and -910, but also at threonine-696 together with impaired signaling by the GTPase RhoA (Zagorska et al., 2010; Banerjee et al., 2014a). MYPT1 phosphoryation at threonine-696 by RhoA/Rho kinase is well-known as a critical step of smooth muscle contraction, in probably any smooth muscle-rich organ (Hennenberg et al., 2008; Hennenberg et al., 2014). In parallel, NUAKs promote formation of actin filaments, consequently regulating actin-dependent functions including cell adhesion and migration (Faisal et al., 2021). In different cell types, including fibroblasts, NUAKs promote proliferation (Faisal et al., 2021). With HTH01-015 and WZ4003, two presumed NUAK inhibitors became available, which both inhibit MYPT phosphorylation and proliferation (Banerjee et al., 2014a). HTH01-015 preferentially inhibits NUAK1, while WZ4003 inhibits both isoforms (Banerjee et al., 2014a). Here, we examined effects of NUAK1 and -2 silencing, and of both inhibitors on growth-related functions of prostate stromal cells, and on contraction of human prostate tissues and of stromal cells.

## 2 Materials and methods

### 2.1 Cell culture

Experiments were carried out in WPMY-1 cells, a SV40 large-T antigen-immortalized line from the stroma of a human prostate without prostate cancer (Webber et al., 1999). According to the typical composition of the prostate stroma, where smooth muscle cells are the predominant cell type, WPMY-1 cells showed characteristics of myofibroblasts and of prostate smooth muscle cells in previous studies, including expression of vimentin, α-smooth muscle actin, calponin and α_1A_-adrenoceptors, but lacking expression of cytokeratins and tyrosine hydroxylase (Webber et al., 1999; Wang et al., 2015; Wang et al., 2016), so that an authentication was here not repeated again. Accordingly, WPMY-1 cells are suitable to examine the impact of knockouts or silencings on prostate smooth muscle contractions (Wang et al., 2021). WPMY-1 cells (catalog no. CRL-2854, RRID:CVCL_3814) were purchased from the American Type Culture Collection (ATCC; Manassas, VA, United States), and cultured in RPMI 1640 (Gibco, Carlsbad, CA, United States) supplemented with 10% fetal calf serum (FCS) and 1% penicillin/streptomycin at 37°C with 5% CO_2_. The medium was changed to a FCS-free medium 24 h prior to the addition of HTH01-015, WZ4003 or dimethylsulfoxid (DMSO, solvent for both inhibitors) to cells.

### 2.2 Human prostate tissues

Human prostate tissues were obtained from patients who underwent radical prostatectomy for prostate cancer. Patients with previous transurethral resection of the prostate were excluded. This study was carried out in accordance with the Declaration of Helsinki of the World Medical Association and has been approved by the ethics committee of the Ludwig-Maximilians University, Munich, Germany (approval number 20-624). Informed consent was obtained from all patients. All samples and data were collected and analyzed anonymously. Accordingly, no patients data were analyzed or related with sampled tissues, such as age, weight, or from medical history. Following removal of prostates from patients, macroscopic pathologic examination and sampling were performed within approximately 30 min. Organ bath experiments, and incubation experiments for Western blot analyses were started within 1 h following sampling. Tissues for Western blot analyses without incubation were shock frozen in liquid nitrogen directly after arrival in the laboratory. For transport and storage, organs and tissues were stored in Custodiol^®^ solution (Köhler, Bensheim, Germany). For macroscopic examination and sampling of prostate tissues, performed by pathologists, the prostate was opened by a single longitudinal cut reaching from the capsule to the urethra. Subsequently, both intersections were checked macroscopically for any obvious tumor infiltration. Tissues were taken from the transitional, periurethral zone, considering that most prostate tumors are located to the peripheral zone (Pradidarcheep et al., 2011; Shaikhibrahim et al., 2012). In fact, tumor infiltration in the periurethral zone (where sampling was performed) was very rare (less than 1% of prostates). Prostates showing tumors in the periurethral zone upon macroscopic inspection were excluded. BPH is present in ca. 80% of patients with prostate cancer (Alcaraz et al., 2009; Orsted and Bojesen, 2013). The age of patients undergoing radical prostatectomy at our department averages out at 66 ± 7 years (Grabbert et al., 2018), when the prevalence of histological BPH ranges between 60% and 70% (Lepor, 2004). Typically, most tissues previously sampled under the same conditions showed a prostate-characteristic architecture with composition of glands, surrounded by smooth muscle-rich stroma (Wang et al., 2015; Wang et al., 2016). For incubation with HTH01-015 for subsequent Western blot analyses, each tissue was cut into either four or six small pieces (approximately 4 × 2 × 2 mm). Pieces from each prostate were allocated to a control group without inhibitor, and to a HTH01-015 group, without that tissues were pooled (i.e., paired samples from one prostate were examined in each independent experiment). For incubation, tissues were transferred to six well plates, containing 10 mL Custodiol^®^ solution per well. DMSO, HTH01-015 or WZ4003 were added, and plates were incubated at 37°C under slight, continous shaking, for 30 min for analyses of p-MYPT and p-MLC, and for 5 h or 22 h (the latter together with 50 nM dihydrotestosterone, DHT) for analyses of Ki-67, calponin and cytokeratin-19. Finally, tissues were shock frozen in liquid nitrogen, and stored at −20°C until processed as described below.

### 2.3 Transfection of WPMY-1 cells with siRNA

WPMY-1 cells were transfected with scramble siRNA (Silencer^®^ Select scrambled Negative Control siRNA duplex, 4390843), human NUAK1 siRNA (Silencer^®^ Select predesigned siRNA, s90, sequence CAC​UCU​UGU​UUA​UGG​AAC​Att, locus ID 9891, lot ASO2GTFJ), human NUAK2 siRNA (Silencer^®^ Select predesigned siRNA, s37779, sequence CAA​CCA​CCC​UCA​CAU​CAU​Utt, locus 81788, lot ASO2GTFK) (all from Ambion Silencer Select library, Life Technologies, Carlsbad, CA, United States). NUAK and scramble siRNAs were diluted in Opti-MEM (110 pmoles siRNA in every 200 μL, providing final concentrations of siRNA in the cell culture medium of 50 nM). Subsequently, Stromal Cell (PrSC) Avalanche™ Transfection Reagent (EZ Biosystems, College Park, MD, United States) was added (1:40) to the Opti-MEM. Cells were 60% confluent at the time of transfection, and were cultured without antibiotics 24 h prior to transfection. Following drop-wise addition of the transfection mixture, plates were centrifuged at 300 *g* for 5 min, incubated at 37°C (5% CO_2_) for 5 h, before the medium was replaced by normal fresh RPMI antibiotic-containing medium.

### 2.4 RT-PCR

RNA isolation, reverse transcription and RT-PCR were performed as previously described (Spek et al., 2021; Wang et al., 2021). Ready-to-use primers were purchased from Qiagen (Hilden, Germany), based on the RefSeq accession numbers NM_014840 for NUAK1, NM_030952 for NUAK2, NM_001299 for calponin-1, NM_002276 for keratin-19, NM_000360 for tyrosin hydroxylase (TH), NM_001030047 for KLK3 (synonymous prostate-specific antigen, PSA), NM_001145966 for Ki-67 and NM_001256799 for GAPDH. Results were expressed using the ∆∆Ct method, where number of cycles (Ct) at which the fluorescence signal exceeded a defined threshold for GAPDH was subtracted from Ct values for targets (Ct_target_-Ct_GAPDH_ = ∆Ct), and values were calculated as 2^−ΔΔCT^.

### 2.5 Western blot analysis

Protein isolation from cells (72 h after transfection, prepared in 75 cm^2^ flasks), homogenization of frozen prostate tissues, protein determination, denaturation with sodium dodecyl sulfate (SDS) sample buffer, electrophoresis, blotting and detection were performed as previously described (Spek et al., 2021; Wang et al., 2021). Boiled lysates corresponding to 30 µg of protein were subjected to SDS polyacrylamide gel electrophoresis. Detection was performed using rabbit anti NUAK1 antibody (MBS9202740) (MyBioSource, San Diego, CA, United States), rabbit anti NUAK2 antibody (ABIN3021383) (antibodies-online, Aachen, Germany), rabbit anti phospho-MLC (Thr18/Ser19) antibody (#3674, RRID:AB_2147464), rabbit monoclonal anti MLC antibody (#8505, RRID:AB_2728760), sheep anti phospho-MYPT (Ser445) antibody (CABT-BL6360, Creative Diagnostics, New York City, NY, United States), rabbit anti phospho-MYPT1 (Ser472) antibody (#PA5-114608, RRID:AB_2899244, Thermo Fisher, Waltham, MA, United States), rabbit anti phospho-MYPT1 (Thr696) antibody (#5163, RRID:AB_10691830), rabbit monoclonal anti MYPT1 antibody (#8574, RRID:AB_10998518) (all from Cell Signaling Technology, Danvers, MA, United States), or mouse monoclonal anti β-actin antibody (sc-47778, RRID:AB_626632) (Santa Cruz Biotechnology, Santa Cruz, CA, United States) for 90 min. Primary antibodies were diluted 1:1000 in phosphate-buffered saline containing 0.1% Tween 20 (PBS-T) and 5% milk powder, with the exeption of the anti β-actin antibody (1:600). Incubation with secondary antibodies was performed with peroxidase-coupled goat anti rabbit IgG (H&L) (#401315, RRID:AB_437787) or with peroxidase-coupled goat anti mouse IgG (H&L) (#401215, RRID:AB_10682749) (both from Merck, Darmstadt, Germany), both diluted 1:3,000 in PBS-T containing 5% milk powder, for detection of NUAK1 and β-actin. For detection of all other antigens, incubation with secondary antibodies was performed using biotinylated goat anti rabbit IgG (BA-1000, RRID:AB_2313606) (Vector Laboratories, Burlingame, CA, United States) diluted 1:1,500 in PBS-T containing 5% milk powder). Intensities of presumed NUAK1 and NUAK2 bands and bands for calponin, keratin-19, MLC, phospho-MLC, MYPT1, phospho-MYPT1 and β-actin were quantified densitometrically using “ImageJ” (National Institutes of Health, Bethesda, Maryland, United States), and referred to β-actin in corresponding samples. For calculation of percentage decreases, values from samples with NUAK-specific siRNA or with inhibitors were referred to means of corresponding control samples. Representative blots in figures were not vertically cropped, i.e., all shown bands are in the same, original order as the samples in gels and on membranes.

### 2.6 Contraction assay

Contractility of cells was measured using the Floating Matrix Model version of the CytoSelect™ 24-Well Cell Contraction Assay Kit (Cell Biolabs, San Diego, CA, United States). Cells were cultured in 75 cm^2^ flasks for a total of 72 h in experiments with silenced cells or for a total of 48 h for experiments with inhibitors, before being trypsinized, resuspended in fresh RPMI medium to a dilution of 10^7^ cells/mL and being transferred to 24-well plates for contraction assays. Transfection was perfomed 48 h before trypsinization as described above, while inhibitors were added 24 h before trypsinization. Transfer to matrix plugs and contraction assays were performed as previously described (Wang et al., 2021). For monitoring of collagen contraction, pictures were taken 1, 2, 3, 6, 12, 24 or 48 h after adding of RPMI (corresponding to 2-49 h after matrix plug filling). Diameters of the collagen plugs and wells on pictures were quantified using ImageJ (RRID:SCR_003070), and contractions were calculated and expressed as (well diameters)—(plug diameters at given time points) in mm.

### 2.7 Assessment of Ki-67 content

Ki-67 mRNA has been commonly assessed to monitor proliferation in various cell types, including WPMY-1 cells (Wang et al., 2021), and was assessed by RT-PCR. Transfected cells were grown for 48 h in 6-well plates before mRNA isolation. HTH01-015, WZ4003 or DMSO were added as 24 h before RNA isolation. Prostate tissues were incubated in 6-well plates containing Custodiol^®^ solution as decribed above. RNA isolation from cells and tissues, and RT-PCR were performed as decribed above. Results are expressed as 2^−ΔCt^ (as described above).

### 2.8 Proliferation assay

Proliferation rate of cells was assessed using the 5-ethynyl-2’-deoxyuridine- (EdU-)based EdU-Click 555 proliferation assay kit (Baseclick, Tutzing, Germany), as previously described (Wang et al., 2021), but with following modifications. EdU stock solution was added 24 h after placing 10,000 cells per well in coverslips. Fixation of transfected cells (and non-transfected cells included to this series) was performed after further 24 h, corresponding to determination of proliferation rate 48 h after transfection. To assess effects of HTH01-015 and WZ4003, again 10,000 cells were placed per well, and cultured for 24 h in FCS-free medium, before inhibitors or DMSO were added. After incubation for further 24 h, the medium was replaced by 10 mM EdU solution in FCS-free smooth muscle cell medium containing inhibitors or DMSO and cultured for another 12 h before fixing, corresponding to determination of proliferation rate 36 h after addition of inhibitors or DMSO. Stained nuclei were counted using “ImageJ” (National Institutes of Health, Bethesda, Maryland, United States), and the rate of proliferating cells (%) was calculated as percentage of EdU-stained nuclei from all nuclei (=EdU- and DAPI-stained).

### 2.9 Cell apoptosis and cell death analysis

A flow cytometry-based annexin V allophycocyanin (APC) and 7-aminoactinomycin D (7-AAD) apoptosis detection kit (BD Biosciences, Franklin Lakes, NJ, United States) was used to detect apoptotic (annexin V-positive, 7-AAD-negative) and dead (annexin V-positive, 7-AAD-positive) cells. Cell death in annexin V-positive/7-AAD-positive cells may result from apoptosis or necrosis, which can not be distinguished by this assay. For comparisons between transfected cells (and non-transfected cells cultured under the same conditions), cells of different groups were grown and transfected in 6-well plates. 48 h after transfection, the remaining medium of each well was collected and centrifuged to collect the cell debris. Then, cells were washed with PBS and resuspended (together with the corresponding debris collected) in annexin V binding buffer (BD Biosciences), followed by addition of 5 μL APC annexin V and 5 μL 7-AAD reagent to every 10^5^ cells from each sample. After incubation in the dark for 15 min at room temperature, 400 μL binding buffer were added to each sample before analysis by flow cytometry. To assess effects of inhibitors, the procedure was the same, but HTH01-015, WZ4003 or DMSO were added 24 h before flow cytometry.

### 2.10 Viability assay

Viability was assessed using the Cell Counting Kit-8 (CCK-8) (Sigma-Aldrich, Munich, Germany). For comparisons of proliferation rates between transfected cells (and non-transfected cells cultured under the same conditions), cells were seeded in 96-well plates (5,000 cells/well) and transfected with siRNA (NUAK-specific or scramble), followed by culturing for 24 h. Finally, 10 μL of [2-(2-methoxy-4-nitrophenyl)- 3-(4-nitrophenyl)-5-(2,4-disulfophenyl)-2H-tetrazolium monosodium salt (WST-8) from the kit were added, and absorbance in each well was measured at 450 nm after incubation for 2 h at 37°C and results are expressed as optical densities (OD). To assess effects of inhibitors, cells were cultured and prepared as described above but without transfection, and cells were cultured for 24, 48 or 72 h with HTH01-015, WZ4003.

### 2.11 Colony formation assay

To assess effects of inhibitors, HTH01-015, WZ4003 or solvent were added 24 h after transfer of cells to 6-well plates (100 cells/well), followed by culture of further 13 days. Finally, plates were washed five times with cold water, stained with 0.4% sulforhodamine B solution at room temperature for 30 min, and washed five times with 1% acetic acid before capturing photos. Visible colonies from each well were counted, and results are expressed as number of colonies per well.

### 2.12 Phalloidin staining

For phalloiding staining of polymerized actin, 10,000 cells of each group were placed per well of 16-well chambered coverslips (Thermo Scientific, Waltham, MA, United States), followed by transfection with siRNAs, or by addition of inhibitors or solvent, and cultured in FCS-free medium. 48 h after transfection, or 24 h or 48 h after addition of inhibitors or solvent, cells were fixed, stained and microscoped as previously described (Wang et al., 2021). Finally, all stainings were quantified using “ImageJ” (National Institutes of Health, Bethesda, Maryland, United States), and phalloidin-stained areas were expressed as percentage of whole areas per microscopic field.

### 2.13 Organ bath

Prostate strips (6 × 3 × 3 mm) were mounted in organ baths (Danish Myotechnology, Aahus, Denmark) with four chambers, stretched to 4.9 mN, equilibrated, and contracted by 80 mM KCl as previously described (Huang et al., 2022). Following washout of KCl, HTH01-015, WZ4003 or equivalent amounts of solvent for controls were added. Cumulative concentration response curves for α_1_-adrenergic agonists, U46619 or endothelin-1, or frequency response curves for electric field stimulation (EFS) were constructed 30 min after addition of inhibitors or solvent. EFS induces neurogenic contractions (Spek et al., 2021), and was applied as previously described (Huang et al., 2022).

Each independent experiment was performed using tissue from the same prostate, which was examined with one inhibitor and as control group (DMSO). For randomization, allocations of drug and control channels were changed between experiments. Blinding was not feasible because experiments were performed by the same experimenters who weighed, dissolved and diluted the drugs. Only one concentration response or frequence response curve was recorded with each tissue. Wherever possible, double determinations were performed. For double determinations, two from the four organ bath channels (filled with tissue from the same prostate) were examined with inhibitor (either HTH01-015, or WZ4003), and the two others with DMSO. From a total of 128 organ bath experiments, double determinations in both groups were possible in 68 experiments. In the remaining experiments, the amount of sampled tissues did not allow filling of two channels for both groups, so that single determinations were peformed in one group (in 28 out of 128 experiments), or in both groups (in 32 out of 128 experiments). However, each experiment contained at least one sample for both groups, resulting in paired samples. For calculation of agonist- and EFS-induced contractions, tensions were expressed as percentage of maximum 80 mM KCl-induced contractions, as this may correct varying phenotypes and degrees of BPH, or any individual variation and heterogeneity between prostate samples and patients.

E_max_ values, EC_50_ values for contractile agonists, and frequencies (f) inducing 50% of the maximum EFS-induced contraction (Ef_50_) were calculated separately for each single experiment by curve fitting as previously described (Huang et al., 2022) using GraphPad Prism 6 (RRID:SCR_002798) (Statcon, Witzenhausen, Germany), and analyzed as described below. Values were checked for plausibility and settings were adapted as follows if error messages occured, as recommended in the “GraphPad Curve Fitting Guide” (GraphPad Software Inc., San Diego, CA, United States). Error messages where most common with U46619, where they occured in eight out of a total of 20 experiments. In these experiments, values at one to two concentrations were omitted, as curves could otherwise not be converged (i.e., curve fitting was not possible, due to downhill parts at higher concentrations), or values were marked as “ambigous” by the programm. In few other experiments (one with phenylephrine and 500 nM WZ4003, one with EFS and 500 nM HTH01-015, two with endothelin-1 and 500 nM HTH01-015, one with noradrenaline and 10 µM HTH01-015), results were unplausible or marked by error messages, while it was not possible to adapt this as described above. In these experiments, E_max_ values and EC_50_/Ef_50_ values were replaced by values at the highest applied concentration or frequence.

### 2.14 Drugs and nomenclature

HTH01-015 (5,13-Dihydro-4,5,13-trimethyl-2-[[1-(4-piperidinyl)-1H-pyrazol-4-yl]amino]-6H-naphtho(2,3-e)pyrimido(5,4-b)(1,4)diazepin-6-one) and WZ4003 (N-3-[[5-Chloro-2-[[2-methoxy-4-(4-methyl-1-piperazinyl)phenyl]amino]-4-pyrimidinyl]oxy]phenyl]propanamide) are structurally related NUAK inhibitors of the N-phenylpyrimidin-2-amine series (Banerjee et al., 2014a). Stock solutions (10 mM) and dilutions were prepared in DMSO, and aliquots were stored at −20°C until use. Both were obtained from Tocris (Bristol, United Kingdom). Phenylephrine ((R)-3-[-1-hydroxy-2-(methylamino)ethyl]phenol) and methoxamine (α-(1-aminoethyl)-2,5-dimethoxybenzyl alcohol) are α_1_-selective adrenoceptor agonists. Aqueous stock solutions (10 mM) of noradrenaline, phenylephrine, and methoxamine were freshly prepared before each experiment. Aqueous stock solutions of endothelin-1 (0.4 mM) were stored at −20°C as small aliquots, so that repeating freezing and thawing cycles were avoided. U46619 ((Z)-7-[(1S,4R,5R,6S)-5-[(E,3S)-3-hydroxyoct-1-enyl]-3-oxabicyclo[2.2.1]heptan-6-yl]hept-5-enoic acid) is an agonist of the thromboxane A_2_ receptor and was dissolved in ethanol. Stock solutions (10 mM) were stored at −80°C until use. U46619 and endothelin-1 were obtained from Enzo Life Sciences (Lörrach, Germany). Noradrenaline, phenylephrine, methoxamine and 5α-dihydrotestosterone (DHT) were obtained from Sigma-Aldrich (Munich, Germany).

### 2.15 Data and statistical analyses

Data from cell culture experiments, RT-PCR, Western blot analyses and curve fitting of organ bath experiments (E_max_, EC_50_ and Ef_50_ values) are shown as scatter plots, including all single values from each independent experiment, together with means and representative experiments if applicable. Data in frequency and concentration response curves are means ± standard deviation (SD). Single values from organ bath experiments and from RT-PCR are based on double determinations, as far as applicable as described above. Obvious effects seen in cell culture and incubation experiments are reported as percent decreases or fold-increases in the text, calculated by reference of each single value from the treatment groups (NUAK-specific siRNAs, or inhibitor groups) to the mean of the corresponding control group. Effects seen in concentration or frequency response curves and on E_max_ values are reported as percent decreases in the text, calculated by reference of values from the inhibitor group to the corresponding sample in the control group. Changes in EC_50_ values are reported in the text as mean differences (MD) with 95% confidence intervals (CIs).

Calculation of MDs and 95% CIs, and all statistical tests were performed using GraphPad Prism 6. Derived from our experimental design, control and drug groups were paired in each series. Comparison of whole frequency/concentration response curves, i.e., of both groups within a series was performed by two-way analysis of variance (ANOVA). Posthoc analyses for comparisons at single agonist concentrations or frequencies were not performed, as this has been discouraged by the “GraphPad Statistics Guide” (GraphPad Software Inc., San Diego, CA, United States). E_max_, Ef_50_, and EC_50_ values were compared by a paired Student’s *t*-test. Multiple comparisons in data sets including non-transfected (“wildtype”), scramble siRNA-transfected and NUAK siRNA-transfected cells were performed by one-way ANOVA with Tukey’s multiple comparisons test. Multiple comparisons in data sets includung a control group and more than one concentration of inhibitor were performed by one-way ANOVA with Dunnett’s tests. *p* values < 0.05 were considered significant. Despite definite aims and a study plan, the present study and analyses have exploratory character, and were not designed to test a pre-specified or statistical null hypothesis. In fact, important features of hypothesis-testing studies are lacking, including definition of a tested hypothesis, blinding or a study plan based on biometric calculation of groups sizes by power analyses (Michel et al., 2020). Owing to the exploratory study design, *p* values reported here need to be considered as descriptive, and are not hypothesis-testing (Michel et al., 2020). In order to use *p* values sparingly, no *p* values are reported in the text and *p* values of 0.05 or higher are not indicated.

The minimum number of independent experiments in each series was pre-planned as *n* = 5, as statistical analyses with group sizes <5 have been discouraged (Curtis et al., 2015). Cell culture experiments provided conclusive results after five independent experiments and are consequenty based on group sizes of *n* = 5, with the exception of detection of MLC and MYPT for reasons explained below. In organ bath experiments, data were analyzed, after five experiments of a series were completed. Subsequently, a series was discontinued if it became obvious that no effect could be expected, or *p* values < 0.05 were obtained. If results after interim analyses were inconclusive, i.e., pointed to a possible drug effect but without *p* values < 0.05, series were continued and analyzed again. This procedure is possible, as our study was explorative (Michel et al., 2020), and flexible group sizes have been recommended if data are characterized by large variations, what applies here (Curtis et al., 2015). Western blot analyses for NUAK1 and -2 with human prostate tissues was performed with tissues from seven different patients, while RT-PCR for NUAK isoforms and markers was performed with tissues from 20 patients. In all series, no experiments and no data were excluded from analyses. Exceptions from general group sizes of *n* = 5 were experiments for detection of MLC and MYPT1, and tissue incubation for 5-22 h. In particular detection of p-MLC and MLC is notoriously prone to fail, due to high background, highly skewed bands, and/or weak or lacking bands in Western blot analyses. In incubation experiments, in partiuclar using periods longer than 5 h, prostate tissues are prone to decay, limiting their viability, detection of mRNA and by Western blot, and data quality. Consequently, group sizes in these experiments exceeded *n* = 5 in these series.

## 3 Results

### 3.1 Expression and silencing of NUAK isoforms in WPMY-1 cells and human prostate tissues

**FIGURE 1:**
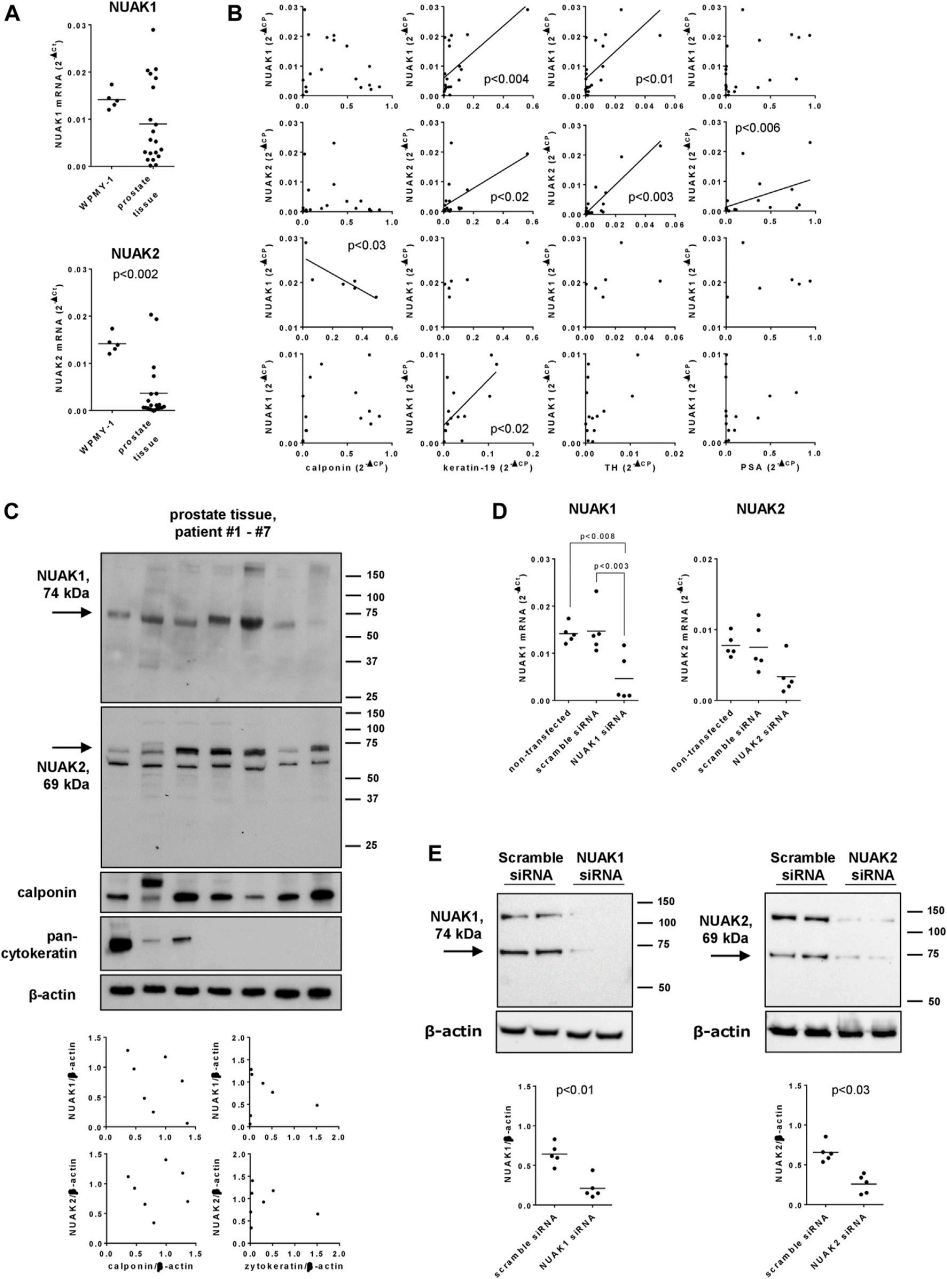
Expression and silencing of NUAK1 and NUAK2 in WPMY-1 cells and human prostate tissues. WPMY-1 cells and human prostate tissues were analyzed for NUAK1 and NUAK2 mRNA by RT-PCR **(A,B,D)**, or by Western blot analysis with antibodies raised against NUAK1 and NUAK2 **(C,E)**. **(A)** Comparison of NUAK1 and NUAK2 mRNA expression between WPMY-1 cells and human prostate tissues. **(B)** Correlation analyses, with mRNA levels of NUAK1, NUAK2, calponin (marker for smooth muscle), keratin-19 (marker for glandular, epithelial cells), tyrosin hydroxylase (TH, marker for catecholaminergic neurons) and prostate-specific antigen (PSA, marker for BPH) in human prostate tissue. Ct values for NUAK1 in prostate tissues showed a binominal distribution, so that groups with high (ddCt >0.01) and low (ddCt <0.01) were analyses separately, in addition to analyses with all examined tissues. **(C)** Western blot analyses of human prostate tissues with NUAK1 and -2 antibodies, and for calponin and pan-cytokeratin. Diagrams show correlation analyses of both NUAK isoforms with calponin and pan-cytokeratin, after quantification of bands in Western blots. Arrows indicate bands with sizes closest to expected molecular weights of NUAKs, which were densitometrically quantified. **(D)** NUAK1 and NUAK2 mRNA in WPMY-1 cells, after transfection with scramble siRNA, or with NUAK1- or NUAK2-specific siRNA. **(E)** Western blot analyses of WPMY-1 cells after transfection with scramble siRNA or siRNAs for NUAK1 or -2, with NUAK1 and -2 antibodies. Arrows indicate bands with sizes closest to expected molecular weights of NUAKs, which were densitometrically quantified. Shown are scatter plots containing single values from each examined sample **(A–E)**, together with means **(A,D,E)**, and with *p* values from Student’s t-test **(A,E)**, Spearman correlation analyses **(B)**, one-way ANOVA with Tukey’s test **(D)** or and together with Western blots including all analyzed tisses **(C)** or representing one representative out of a total of five independent experimts **(E)**.

NUAK1 and NUAK2 mRNA was detectable in human prostate tissues and in WPMY-1 cells (Figure 1A). NUAK1 mRNA levels in human prostate tissues were on average lower as in WPMY-1 cells [0.63 fold (0.349 to 0.919) of mean of WPMY-1], but showed an obvious binominal distribution (Figure 1A), with ddCt values from 14 of 20 examined tissues ranging below 0.01 [range: 0.00016 to 0.0099; 0.43 fold (0.24 to 0.63) of mean in prostate tissues] and ddCt values from 6 tissues ranging above 0.01 [0.017–0.029; 2.3 fold (1.8 to 2.8) of mean in prostate tissues]. Levels of NUAK2 mRNA in prostate tissues were lower as in WPMY-1 cells [0.26 fold (0.06 to 0.46), and did not show a clear binominal distribution (Figure 1A)].

Correlation analyses of NUAK1 and NUAK2 mRNA levels were performed with mRNA levels of calponin as a marker for smooth muscle cells, keratin-19 as a maker for glandular, epithelial cells, tyrosin hydroxylase as a marker for catecholaminergic neurons and PSA as a marker for BPH in human prostate tissues. None of both isoforms correlated with calponin expression, while NUAK1 and NUAK2 both correlated with expression of keratin-19 and tyrosinhydroxylase, and NUAK2 but not NUAK1 with PSA expression (Figure 1B). After separate analyses for subgroups with high (ddCt >0.01) and low (ddCt <0.01) NUAK1 mRNA expression, high NUAK1 expression correlated negatively with calponin expression but not any more with expression of keratin-19 or tyrosinhydroxylase, while low NUAK1 expression still correlated with keratin-19 expression (Figure 1B).

Western blot analyses of human prostate tissues with an antibody raised against NUAK1 revealed single bands with a size close to the expected molecular weight of NUAK1 (74 kDa), which occured with each examined tissue (Figure 1C). Western blot analyses of human prostate tissues with an antibody raised against NUAK2 revealed two bands, one of them with a size close to the expected molecular weight of NUAK2 (69 kDa), and both occuring with each examined tissue (Figure 1C).

Transfection of WPMY-1 cells with NUAK1- or NUAK2-specific siRNA reduced the mRNA expression of the corresponding isoform, compared to cells transfected with scramble siRNA (Figure 1D). After transfection with NUAK1 siRNA, NUAK1 mRNA levels amounted to 0.32 fold (−0.11–0.74) of NUAK1 expression in scramble siRNA-transfected cells, while levels of NUAK2 mRNA after transfection with NUAK2-specific siRNA amounted to 0.447 fold (0.03–0.87) of levels in scramble siRNA-transfected cells (Figure 1D). At protein level, silencing was confirmed by Western blot analyses, where silencing reduced bands migrating close to the expected weights of NUAK1 (74 kDa) and NUAK2 (69 kDa) (Figure 1E). Compared to scramble-transfected controls, intensities of these bands were reduced by 67% (41–93) after transfection with NUAK1 siRNA, and by 61% (38–83) after transfection with NUAK2 siRNA (Figure 1E).

### 3.2 Effects of NUAK silencing, HTH01-015 and WZ4003 on contraction of WPMY-1 cells

**FIGURE 2:**
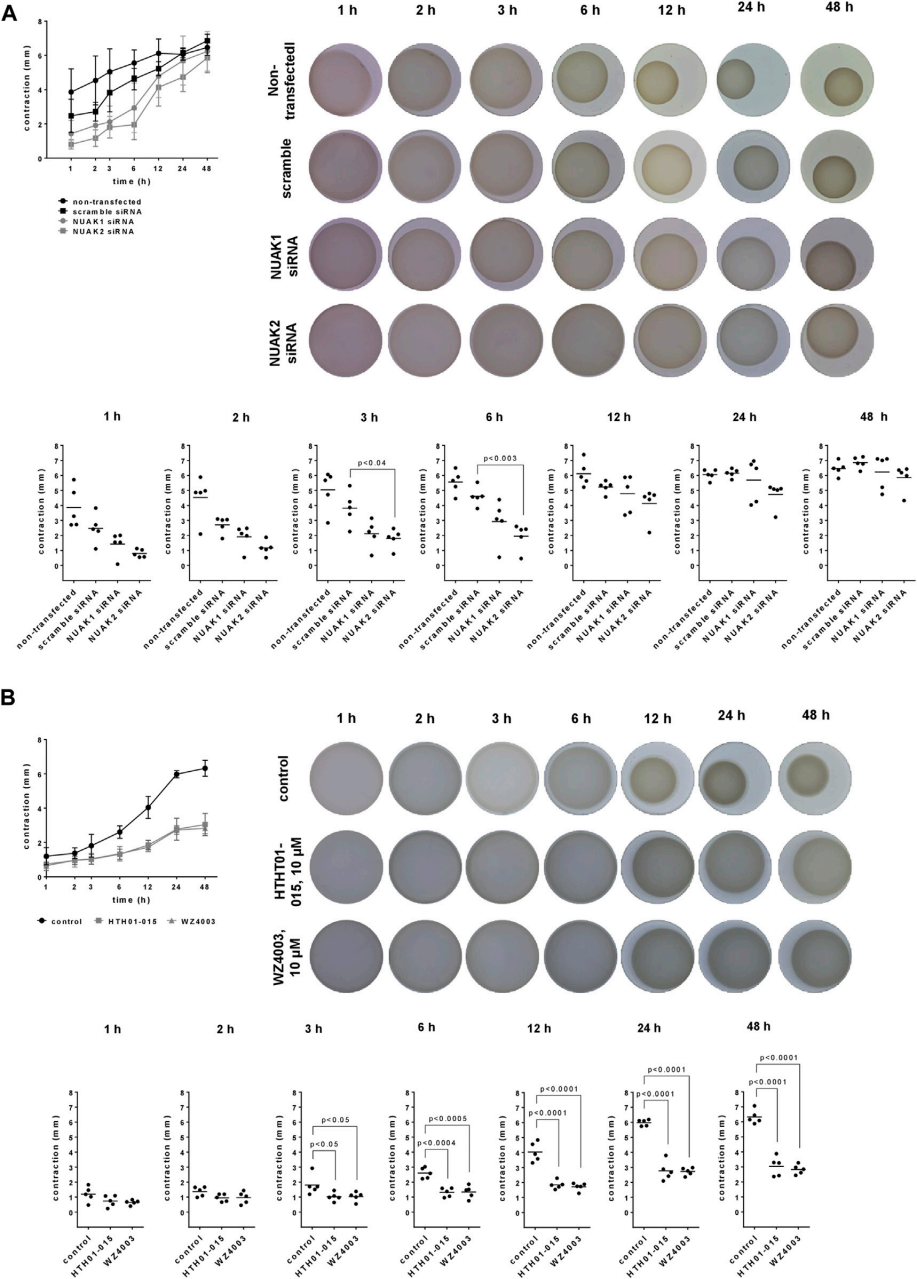
Effects of NUAK silencing, HTH01-015 and WZ4003 on contraction of WPMY-1 cells. Contraction of WPMY-1 cells over time was assessed by matrix contraction, starting 48 h after transfection with scramble siRNA or isoform-specific NUAK siRNAs **(A)**, or starting 24 h after addition of HTH01-015 (10 µM), WZ 4003 (10 µM) or solvent to controls **(B)**. Contractions are expressed as decreases in plug diameter during indicated periods. Shown are contractions over time (means with SD), representative pictures for each condition, and single values from each sample and each single experiment for each time point in scatter plots, including *p* values from one-way ANOVA with Tukey’s test in **(A)** and one-way ANOVA with Dunnett’s test in **(B)**.

Contraction of WPMY-1 cells was assessed by matrix contraction assays, 1–48 h after seeding to matrix plugs. Contractions increased over time, and were lower in cells transfected with NUAK1 or NUAK2 siRNA, compared to contractions in scramble siRNA-transfected cells (Figure 2A). Decreases in contraction by transfection with NUAK-specific siRNAs was largest at 3–6 h after seeding, but virtually lacking after 24–48 h (Figure 2A). Compared to scramble siRNA-transfected cells, contractions were decreased by 45% (15–75) after 3 h and by 36% (−3–75) after 6 h by transfection with NUAK1 siRNA, and by 53% (32–73) after 3 h and by 58% (34–81) after 6 h by transfection with NUAK2 siRNA (Figure 2A).

Compared to solvent-treated controls, contractions of WPMY-1 cells were reduced by HTH01-015 10 µM and WZ4003 (10 µM), to similar degree by both inhibitors and over the complete period from 3 to 48 h after seeding (Figure 2B). Inhibitions were largest at 12–48 h after seeding. Compared to controls, contractions were decreased by 54% (46–63) after 12 h, by 54% (40–67) after 24 h and by 52% (39–65) after 48 h by HTH01-015, and by 57% (49–65) after 12 h, by 54% (49–60) after 24 h and by 55% (49–62) after 48 h by WZ4003 (Figure 2B).

### 3.3 Effects of NUAK silencing, HTH01-015 and WZ4003 on proliferation of WPMY-1 cells

**FIGURE 3:**
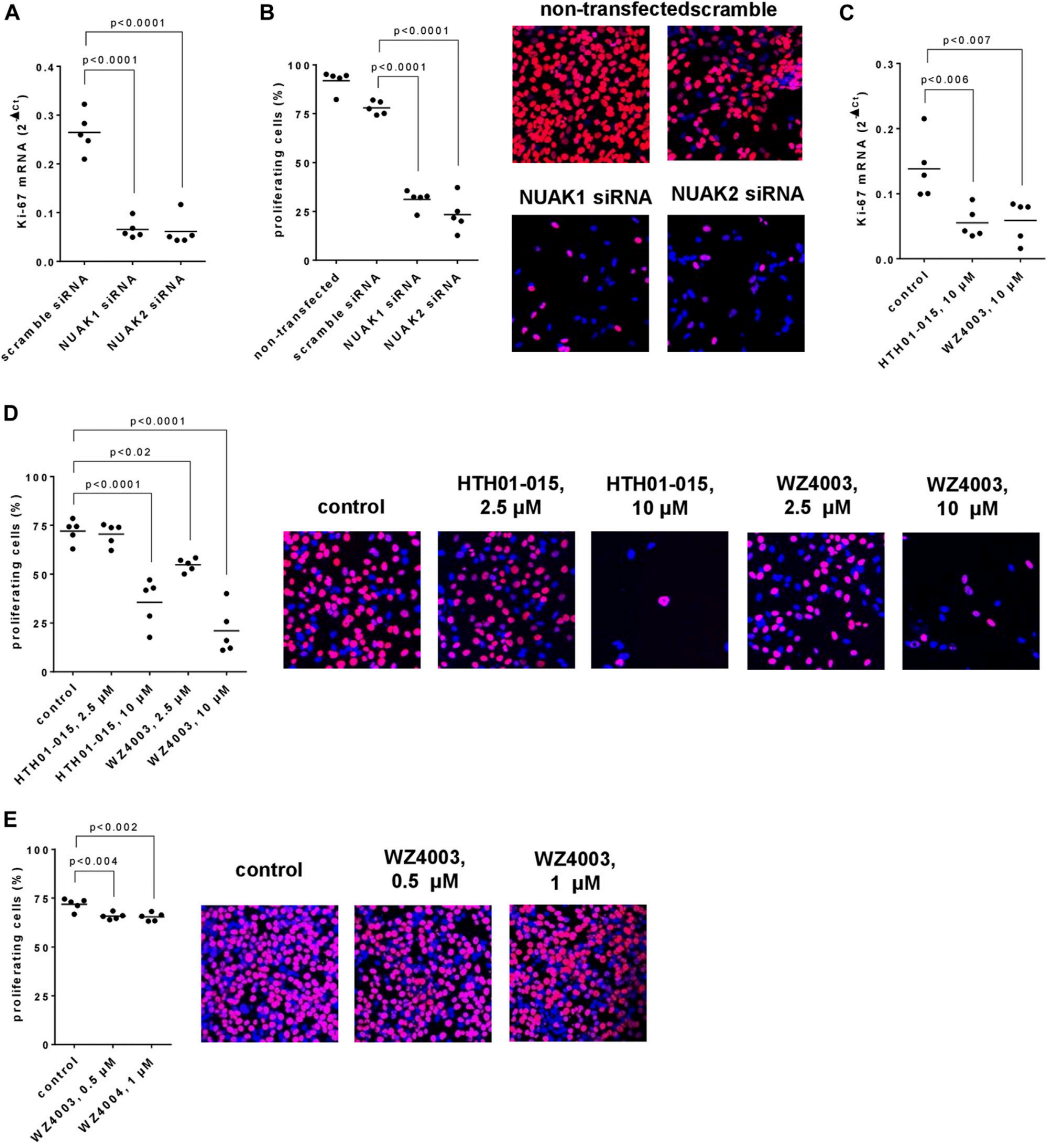
Effects of NUAK silencing, HTH01-015 and WZ4003 on proliferation of WPMY-1 cells. Proliferation was assessed 48 h after transfection with scramble siRNA of isoform-specific NUAK siRNAs **(A,B)**, or 36 h after addition of HTH01-015, WZ4003 or solvent to controls **(C–E)** as the content of Ki-67 mRNA **(A,C)** or by EdU assays **(B,D,E)**. Shown are scatter plots including all single values from each independent experiment together with *p* values from one-way ANOVA with Tukey’s test in **(B)** and one-way ANOVA with Dunnett’s test in all other panels, and representative experiments from EdU assays.

Transfection with NUAK siRNAs resulted in reduced mRNA levels of the proliferation marker Ki-67, assessed 48 h after transfection (Figure 3A). Compared to scramble siRNA-transfected cells, Ki-67 mRNA levels were decreased by 75% (66–84) in cells transfected with NUAK1 siRNA, and by 77% (62–91) in cells transfected with NUAK2 siRNA (Figure 3A). In parallel, the proliferation rate of WPMY-1 cells, assessed bei EdU assays, was reduced by transfection with NUAK1- and NUAK2-specific siRNA (Figure 3B). Compared to scramble siRNA-transfected cells and assessed 48 h after transfections, proliferation rates were decreased by 60% (53–68) in cells transfected with NUAK1 siRNA, and by 70% (56–84) in cells transfected with NUAK2 siRNA (Figure 3B).

HTH01-015 and WZ4003, applied in concentrations of 10 µM and for 24 h, resulted in reduced Ki-67 mRNA levels (Figure 3C). Compared to solvent-treated controls, Ki-67 mRNA levels were decreased by 60% (39–81) by HTH01-015, and by 57% (29–85) by WZ4003 (Figure 3C). In parallel and compared to solvent-treated controls, the proliferation rate of WPMY-1 cells was reduced by 10 μM, but not 2.5 µM HTH01-015, and by 2.5 and 10 µM WZ4003, both applied for 24 h (Figure 3D). Decreases amounted to 51% (30–72) with 10 µM HTH01-015, and to 24% (18–30) with 2.5 µM and 71% (50–92) with 10 µM WZ4003 (Figure 3D). Effects of lower concentrations were examined in a separate series for WZ4003, but not for HTH01-015, as 2.5 µM HTH01-015 failed to reduce proliferation rates. Compared to solvent-treated controls, the proliferation rate was slightly reduced by 0.5 and 1 µM WZ4003, both applied for 24 h (Figure 3E). Decreases amounted to 8% (6–11) with 0.5 µM WZ4003, and to 9% (5–13) with 10 µM WZ4003 (Figure 3E).

### 3.4 Effects of NUAK silencing, HTH01-015 and WZ4003 on apoptosis and cell death of WPMY-1 cells

**FIGURE 4:**
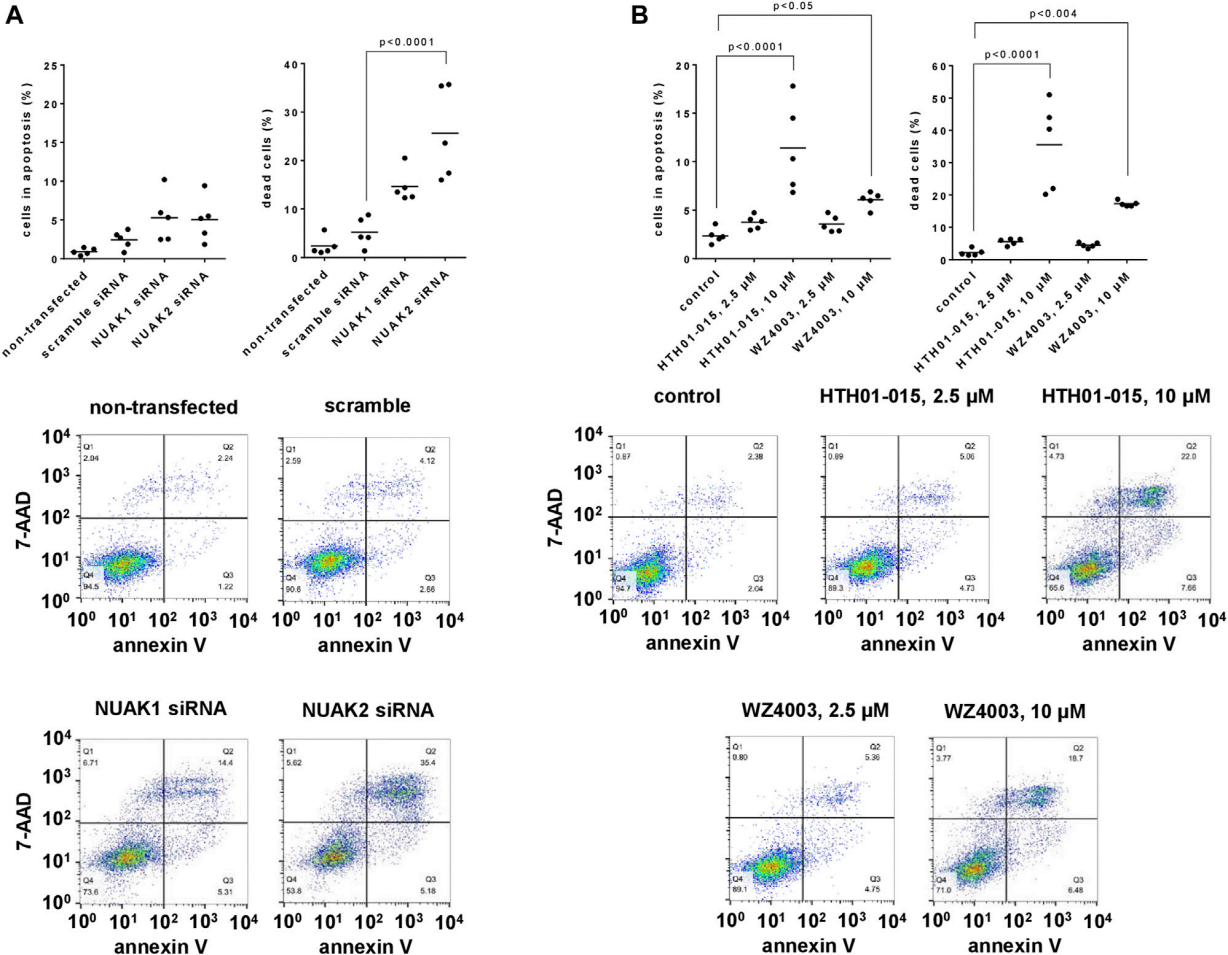
Effects of NUAK silencing, HTH01-015 and WZ4003 on apoptosis and cell death of WPMY-1 cells. Apoptosis and cell death were determined by flowcytometry for annexin V and 7-AAD, 48 h after transfection with scramble siRNA or isoform-specific NUAK siRNAs **(A)**, or 24 h after addition of HTH01-015, WZ4003 or solvent to controls **(B)**. Annexin V-positive/7-AAD-negative cells represent apoptotic cells, wheras annexin V-positive/7-AAD-positive cells represent dead cells. Shown are scatter plots including all single values from each independent experiment together with *p* values from one-way ANOVA with Tukey’s test in **(A)**, and from one-way ANOVA with Dunnett’s test in **(B)**, and representative experiments.

Compared to scramble siRNA-transfected cells and assessed 48 h after transfections, the number of apoptotic cells (annexin V-positive, 7-AAD-negative) was slightly inreased, whereas the number of dead cells (annexin V-positive, 7-AAD-positive) was strongly increases by transfection with NUAK1- or NUAK2-specific siRNA (Figure 4A). The number of apoptotic cells after transfection with NUAK1 or NUAK2 siRNA amounted to 2.2 fold (0.6–3.8) or 2.1 fold (0.6–3.5), respectively, of the number of apoptotic cells after transfection with scramble siRNA (Figure 4A). The number of dead cells after transfection with NUAK1 or NUAK2 siRNA amounted to 2.8 fold (2.0–3.6) or 4.9 fold (2.6–7.2), respectively, of the number of apoptotic cells after transfection with scramble siRNA (Figure 4A).

Using concentrations of 10 µM and applied for 24 h, HTH01-015 and WZ4003 both increased the number of apoptotic and the number of dead cells, while concentrations of 2.5 µM caused no or only minor increases (Figure 4B). Referred to the number of apoptotic cells in solvent-treated controls, the number of apoptotic cells amounted to 1.6 fold (1.2–2.0) with 2.5 µM and 4.9 fold (2.4–7.3) with 10 µM HTH01-015, and to 1.5 fold (1.1–2.0) with 2.5 µM and 2.6 fold (2.1–3.0) with 10 µM WZ4003 (Figure 4B). Referred to the number of dead cells in solvent-treated controls, the number of dead cells amounted to 2.5 fold (2.0–3.1) with 2.5 µM and 16.1 fold (8.4–23.8) with 10 µM HTH01-015, and to 2.0 fold (1.6–2.5) with 2.5 µM and 7.8 fold (7.3–8.3) with 10 µM WZ4003 (Figure 4B).

### 3.5 Effects of NUAK silencing, HTH01-015 and WZ4003 on viability of WPMY-1 cells

**FIGURE 5:**
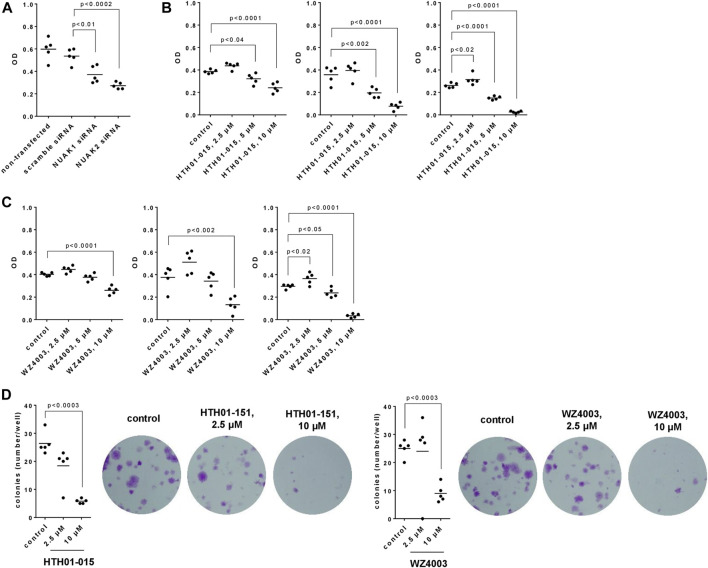
Effects of NUAK silencing, HTH01-015 and WZ4003 on viability and colony formation of WPMY-1 cells. Viability was determined by CCK-8 assays **(A–C)**, and cell growth by colony formation assay **(D)**, 24 h after transfection with scramble siRNA or isoform-specific NUAK siRNAs **(A)**, 24-72 h after addition of HTH01-015, WZ4003 or solvent to controls **(B,C)** or 13 days after addition of HTH01-015, WZ4003 or solvent to controls **(D)**. Shown are scatter plots including all single values from each independent experiment together with *p* values from one-way ANOVA with Tukey’s test in **(A)**, and from one-way ANOVA with Dunnett’s test in **(B–D)**, and representative experiments from colony formation assays.

Transfection with NUAK1- or NUAK2-specific siRNA reduced the viability of WPMY-1 cells in CCK-8 assays, assessed 24 h after transfection (Figure 5A). Compared to scramble siRNA-transfected cells, viabilities were decreased by 31% (13–48) by transfection with NUAK1 siRNA, and by 49% (42–56) by transfection with NUAK2 siRNA (Figure 5A).

HTH01-015 and WZ4003 were applied for 24, 48 or 72 h and caused concentration- and time-dependent decreases of viability (Figures 5B, C). With both inhibitors, decreases were lacking using 2.5 μM at all three incubation periods (24-72 h), occured using 5 μM at each incubation period (HTH01-015) or after 72 h of incubation (WZ4003), and were observed using 10 µM after each incubation period (Figures 5B, C). With 10 μM, the viability was virtually completely reduced after 72 h, corresponding to decreases of 90% (86–94) with HTH01%-015% and 88% (81–96) with WZ4003 (Figures 5B, C).

### 3.6 Effects of HTH01-015 and WZ4003 on colony formation of WPMY-1 cells

The growth of WPMY-1 cells, assessed by colony formation assays, was reduced by HTH01-015 and WZ4003 (Figure 5D). Compared to solvent-treated controls, decreases amounted to 30% (0–61) with 2.5 µM HTH01-015, 78% [74–82) with 10 µM HTH01%-015% and 64% (48–80) with 10 µM WZ4003, while no decreases occured with 2.5 µM WZ4003 (Figure 5D).

### 3.7 Effects of NUAK silencing, HTH01-015 and WZ4003 on actin polymerization

**FIGURE 6:**
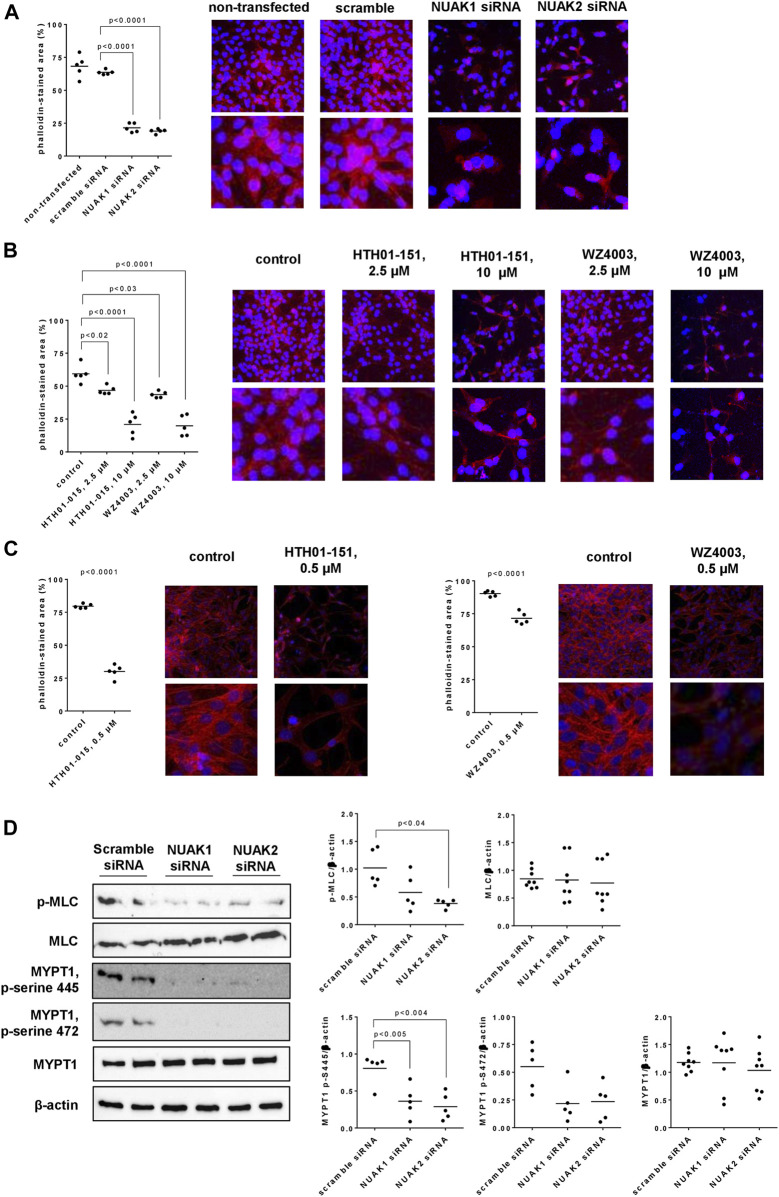
Actin organization and MLC phosphorylation in WPMY-1 cells. Actin organization was assessed by phalloidin staining **(A–C)**, and levels of phospho-MLC, MLC, phospho-MYPT1 and MYPT1 were assessed by Western blot analyses **(D)**. Phalloidin staining was performed 48 h after transfection with scramble siRNA or isoform-specific NUAK siRNAs **(A)**, 24 h after addition of HTH01-015, WZ4003 or solvent to controls **(B)**, or 48 h after addition of HTH01-015, WZ4003 or solvent to controls **(C)**. Western blot analyses were performed with cells transfected with scramble siRNA or siRNAs for NUAK1 and -2 **(D)**. Shown are scatter plots including all single values from quantification of each independent experiment together with *p* values from one-way ANOVA with Tukey’s test in **(A)**, from one-way ANOVA with Dunnett’s test in **(B)**, from Student’s *t*-test in **(C)**, and from one-way ANOVA with Dunnett’s test in **(D)**, and representative experiments from phalloidin staining (pictures in lower panels are cutouts from pictures in upper panels) and representative Western blots.

Silencing of NUAK1 or NUAK2 caused breakdowns in actin polymerization and organization, which was assessed by phalloidin staining 48 h after transfections, and became qualitatively and quantitatively apparent. In cells transfected with scramble siRNA, polymerized (corresponding to phalloidin-stained) actin was organized to elongated, long filaments, with protrusions of different cells overlapping each other (Figure 6A). Transfection with NUAK1- or NUAK2-specific siRNA resulted in an extensive loss of actin filaments. Remaining polymerized actin was centered around nuclei, even though being partly organized to jag-shaped, short protrusions (Figure 6A). Quantification pointed to decreases in polymerized actin of 66% (60–73) by transfection with NUAK1 siRNA, and of 70% (67–73) by transfection with NUAK2 siRNA, compared to scramble siRNA-transfected cells (Figure 6A).

Solvent-treated WPMY-1 cells showed the same actin organization and a similar degree of polymerization as described above for scramble siRNA-transfected cells (Figure 6B). Using concentrations of 2.5 µM (applied for 24 h), HTH01-015 and WZ4003 reduced the content of polymerized actin, without affecting the filament organization of remaining actin (Figure 6B). Remaining filaments were long and thin, and overlapped each other (Figure 6B). Application of 10 µM for 24 h resulted in an extensive loss of actin polymerization with both inhibitors, even though an organization of remaining actin to elongated filaments was still apparent (Figure 6B). Compared to solvent-treated controls, decreases in phalloidin-stained actin amounted to 21% (15–28) with 2.5 µM and 65% (47–82) with 10 µM HTH01-015, and to 27% (21–32) with 2.5 µM and 67% (50–83) with 10 µM WZ4003 (Figure 6B).

As effects were seen with both inhibitors using concentrations of 2.5 µM, effects of a lower concentration (500 nM, applied for 48 h) were examined in separate series (Figure 6C). Compared to solvent-treated controls, phalloidin-stained areas were reduced by 500 nM HTH01-015 or WZ4003, both applied for 48 h (Figure 6C). Decreases amounted to 62% (54–70) with HTH01-015, and to 21% (15–27) with WZ4003 (Figure 6C).

### 3.8 Effects of NUAK silencing on MLC and MYPT1 phosphorylation

Silencing of NUAK1 or NUAK2 reduced the content of phosphorylated MLCs in WPMY-1 cells, without affecting the total MLC content, and were more obvious with NUAK2 silencing than for NUAK1 silencing (Figure 6D). Compared to scramble siRNA-transfected cells, contents of phospho-MLC were decreased by 43% (3–83) by transfection with NUAK1 siRNA, and by 64% (56–73) by transfection with NUAK2 siRNA (Figure 6D). The content of MYPT1 remained unchanged by silencing of NUAK1 or NUAK2, while serine-445 and serine-472-phosphorylated MYPT1 was reduced by silencing of NUAK1 or NUAK2 (Figure 6D), and threonine-696-phosphorylated MYPT1 was undetectable in all groups and samples. Compared to scramble siRNA-transfected cells, contents of serine-445-phosphorylated MYPT1 were decreased by 55% (22–88) by transfection with NUAK1 siRNA, and by 64% (37–92) by transfection with NUAK2 siRNA (Figure 6D). Again compared to scramble controls, contents of serine-472-phosphorylated MYPT1 were decreased by 60% (22–99) by transfection with NUAK1 siRNA, and by 57% (20–94) by transfection with NUAK2 siRNA (Figure 6D).

### 3.9 Effects of HTH01-015 and WZ4003 on expression of Ki-67, calponin and cytokeratin in human prostate tissues

**FIGURE 7:**
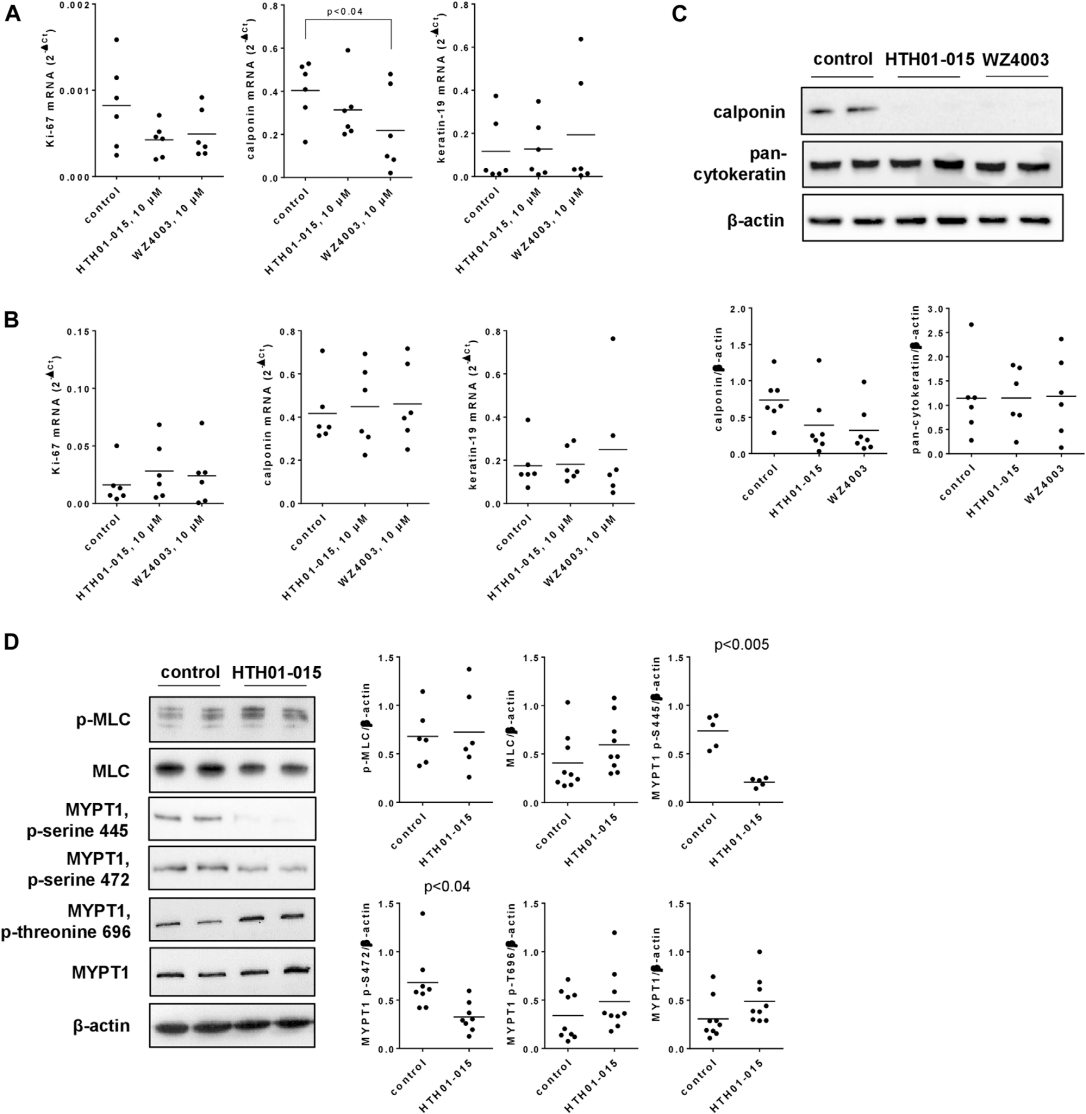
Effects of HTH01-015 and WZ4003 on marker expression, and on MYPT/MLC phosphorylations in human prostate tissues. RT-PCR for Ki-67 (marker for proliferation), calponin (marker for smooth muscle cells) and cytokeratin-19 (marker for glandular epithelial cells) was performed after incubation of human prostate tissues with HTH01-015 WZ4003 (both 10 µM) or solvent for controls for 22 h together with 50 nM DHT (in all samples) **(A)** or 5 h **(B)**. Western blot analyses for calponin and pan-cytokeratin were performed after incubation of human prostate tissues with HTH01-015, WZ4003 (both 10 µM) or solvent for controls for 22 h **(C)**. Western blot analyses for phosphorylated and non-phosphorylated MLC and MYPT1 were performed after incubation of human prostate tissues with HTH01-015 (500 nM) or solvent for controls for 30 min **(D)**. Shown are scatter plots including all single values from quantification of each independent experiment together with *p* values from one-way ANOVA with Dunnett’s test in **(A)**, and Student’s *t*-test in **(D)**, and representative Western blots.

HTH01-015 and WZ4003, applied in concentrations of 10 µM for 22 h to human prostate tissues in the presence of DHT (50 nM), resulted in reduced mRNA levels of Ki-67 and calponin, but not of cytokeratin-19 (Figure 7A). Compared to solvent-treated controls, Ki-67 mRNA levels were decreased by 48% (24–73) by HTH01-015, and by 40% (4–76) by WZ4003 (Figure 7A). In parallel and compared to solvent-treated controls, mRNA levels of calponin, as a marker for smooth muscle cells, was reduced by 22% (−15–60) by HTH01-015, and by 46% (−5–96) by WZ4003 (Figure 7A). Levels of cytokeratin-19, as a marker for glandular epithelial cells, were not reduced by HTH01-015 or WZ4003 (Figure 7A). Application of HTH01-015 or WZ4003 (10 µM) for 5 h to human prostate tissues (without DHT) did not reduce mRNA levels of Ki-67, calponin or cytokeratin-19 (Figure 7B).

Decreases of calponin and unchanged contents of cytokeratin seen at mRNA were confirmed by Western blot at protein level (Figure 7C). Compared to solvent-treated controls, the content of calponin was reduced by 47% (−7–101) by 10 µM HTH01-015, and by 57% (15–98) by 10 µM WZ4003, both applied for 22 h (Figure 7C).

### 3.10 Effects of HTH01-015 on MLC and MYPT1 phosphorylation

Incubation of human prostate tissues with HTH01-015 (500 nM, 30 min) reduced the content of serine-445- and serine-472-phosphorylated MYPT1, but did not afffect the contents of MLC, phospho-MLC, MYPT1 or threonine-696-phosphorylated MYPT1, each assessed by Western blot analyses (Figure 7D). Compared to solvent-treated controls, the content of serine-445-phosphorylated MYPT1 was reduced by 72% (64–79) by HTH01-015, and the content of serine-472-phosphorlyted MYPT1 by 52% (34–71) (Figure 7D).

### 3.11 Effects of HTH01-015 on EFS-induced contractions of human prostate tissues

**FIGURE 8:**
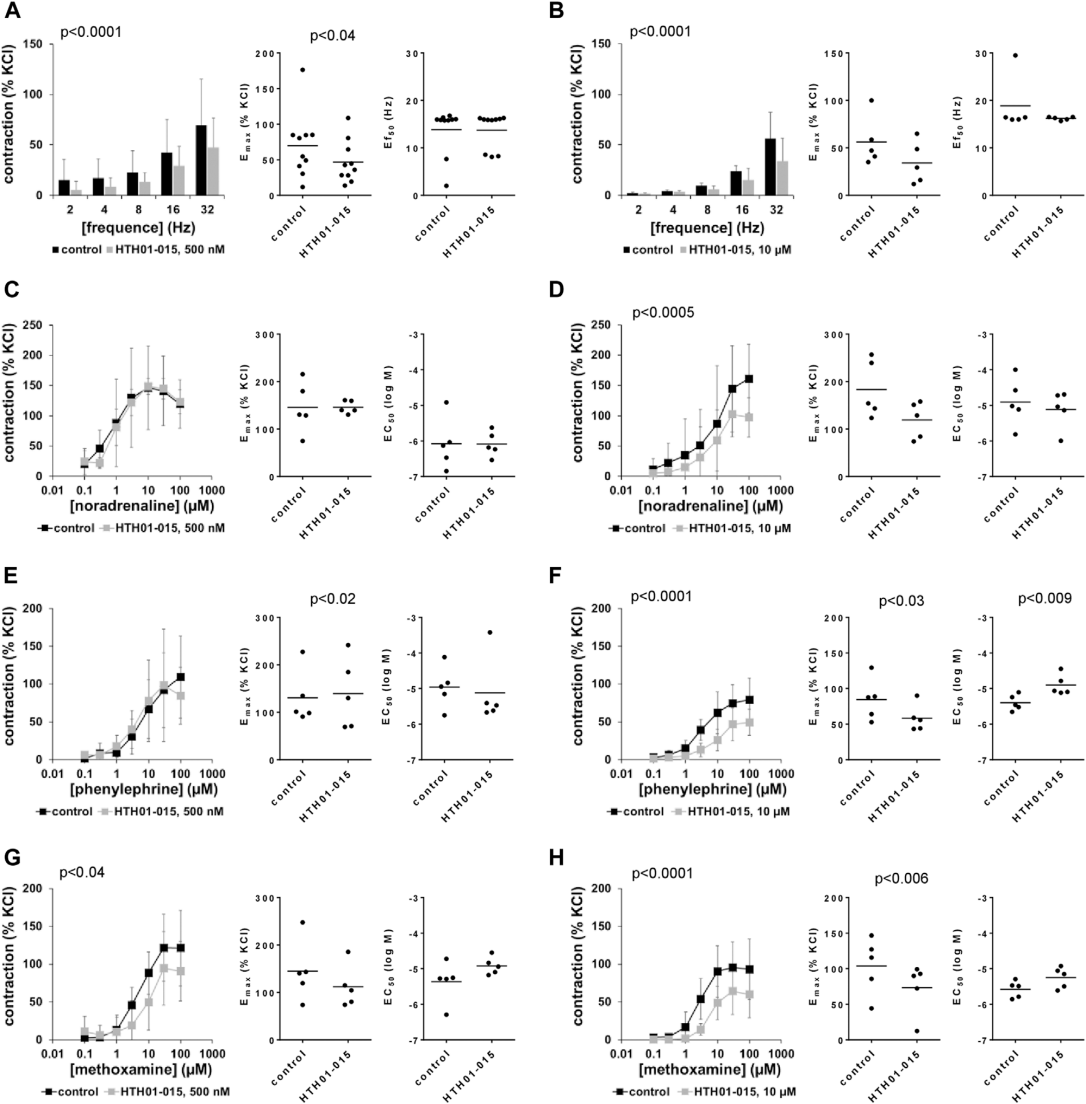
Effects of HTH01-015 on EFS-induced and adrenergic contractions of human prostate tissues. Contractions of human prostate tissues were induced by EFS **(A,B)**, noradrenaline **(C,D)**, phenylephrine **(E,F)** or methoxamine **(G,H)** in an organ bath, 30 min after addition of HTH01-015 in concentrations of 500 nM **(A,C,E,G)** or 10 µM **(B,D,F,H)** or of an equivalent amount of DMSO (controls), which was used as solvent for HTH01-015. Shown are data from *n* = 10 independent experiments in **(A)** and *n* = 5 independent experiments in each other subpanel, where tissues from *n* = 10 or *n* = 5 patients were used for both groups of a subpanel, resulting in paired samples. Shown are means ± SD from all experiments in concentration response curves with *p* values from two-way ANOVA for whole groups, and all single E_max_, EC_50_ and Ef_50_ values from all experiments (calculated by curve fitting) together with *p* values from Student’s *t*-test in scatter plots.

EFS induced frequence-dependent contractions of human prostate tissues, which were reduced by HTH01-015 using concentrations of 500 and 10 µM (Figures 8A, B). Average decreases at 32 Hz, the highest applied frequence amounted to 10% (−47–67) with 500 nM, and to 42% (14–70) with 10 µM HTH01-015. Decreases seen in frequence response curves were reflected by decreases in E_max_ values, amounting to 13% (−45–70) with 500 nM HTH01-015, and to 42% (13–70) with 10 µM HTH01-015 (Figures 8A, B). Ef_50_ values were not affected by HTH01-015 (Figures 8A, B).

### 3.12 Effects of HTH01-015 on adrenergic contractions of human prostate tissues

Noradrenaline, phenylephrine and methoxamine induced concentration-dependent contractions of human prostate tissues, which were not reduced or to small degree by 500 nM HTH01-015, but consistently with all three agonists by 10 µM HTH01-015 (Figures 8C–H). Inhibitions seen in concentration response curves were reflected by decreases in E_max_ values, amouting to decreases by 29% (−10–68) for noradrenaline, 29% (10–47) for phenylephrine and 21% (−46–87) for methoxamine (Figures 8D, F, H). EC_50_ values for all three agonists were not decreased by HTH01-015, but increased around half a magnitude for phenylephrine by 10 µM HTH01-015 (Figures 8C–H).

### 3.13 Effects of HTH01-015 on non-adrenergic contractions of human prostate tissues

**FIGURE 9:**
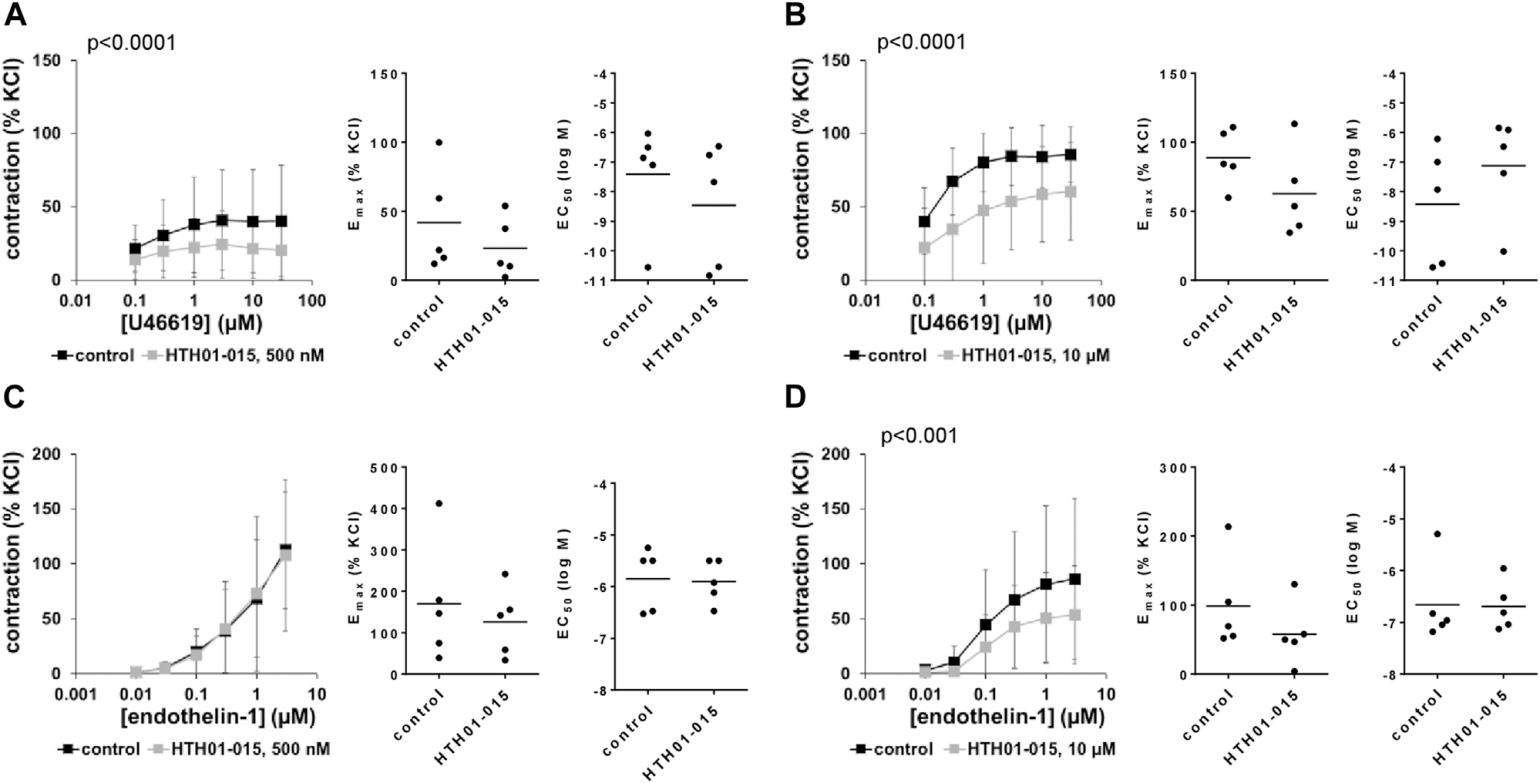
Effects of HTH01-015 on non-adrenergic contractions of human prostate tissues. Contractions of human prostate tissues were induced by U46619 **(A,B)** or endothelin-1 **(C,D)** in an organ bath, 30 min after addition of HTH01-015 in concentrations of 500 nM **(A,C)** or 10 µM **(B,D)** or of an equivalent amount of DMSO (controls), which was used as solvent for HTH01-015. Shown are data from *n* = 5 independent experiments in each subpanel, where tissues from *n* = 5 patients were used for both groups of a subpanel, resulting in paired samples. Shown are means ± SD from all experiments in concentration response curves with *p* values from two-way ANOVA for whole groups, and all single E_max_ and EC_50_ values from all experiments (calculated by curve fitting) in scatter plots.

U46619 and endothelin-1 induced concentation-dependent contractions of human prostate tissues. U46619-induced contractions were inhibited by 500 and 10 µM HTH01-015, with an apparently larger inhibition using 10 µM (Figures 9A, B). Decreases seen in concentration response curves were reflected by decreases in E_max_ values, amounting to 49% (27–71) with 500 nM HTH01-015, and to 30% (−2–63) with 10 µM HTH01-015 (Figures 9A, B). EC_50_ values for U46619 were increased by more than one magnitude (MD (logM) 1.3 (−1.0–3.6) by 10 µM HTH01-015 (Figure 9B), but remained unchanged by 500 nM HTH01-015 (Figure 9A).

Endothelin-1-induced contractions were inhibited by 10 μM, but not by 500 nM HTH01-015 (Figures 9C, D). Decreases seen by 10 µM in concentration response curves were reflected by decreases in E_max_ values, amounting to 37% (−13–88) (Figure 9D). EC_50_ values for endothelin-1 were not affected by WZ4003 (Figures 9C, D).

### 3.14 Effects of WZ4003 on EFS-induced contractions of human prostate tissues

**FIGURE 10:**
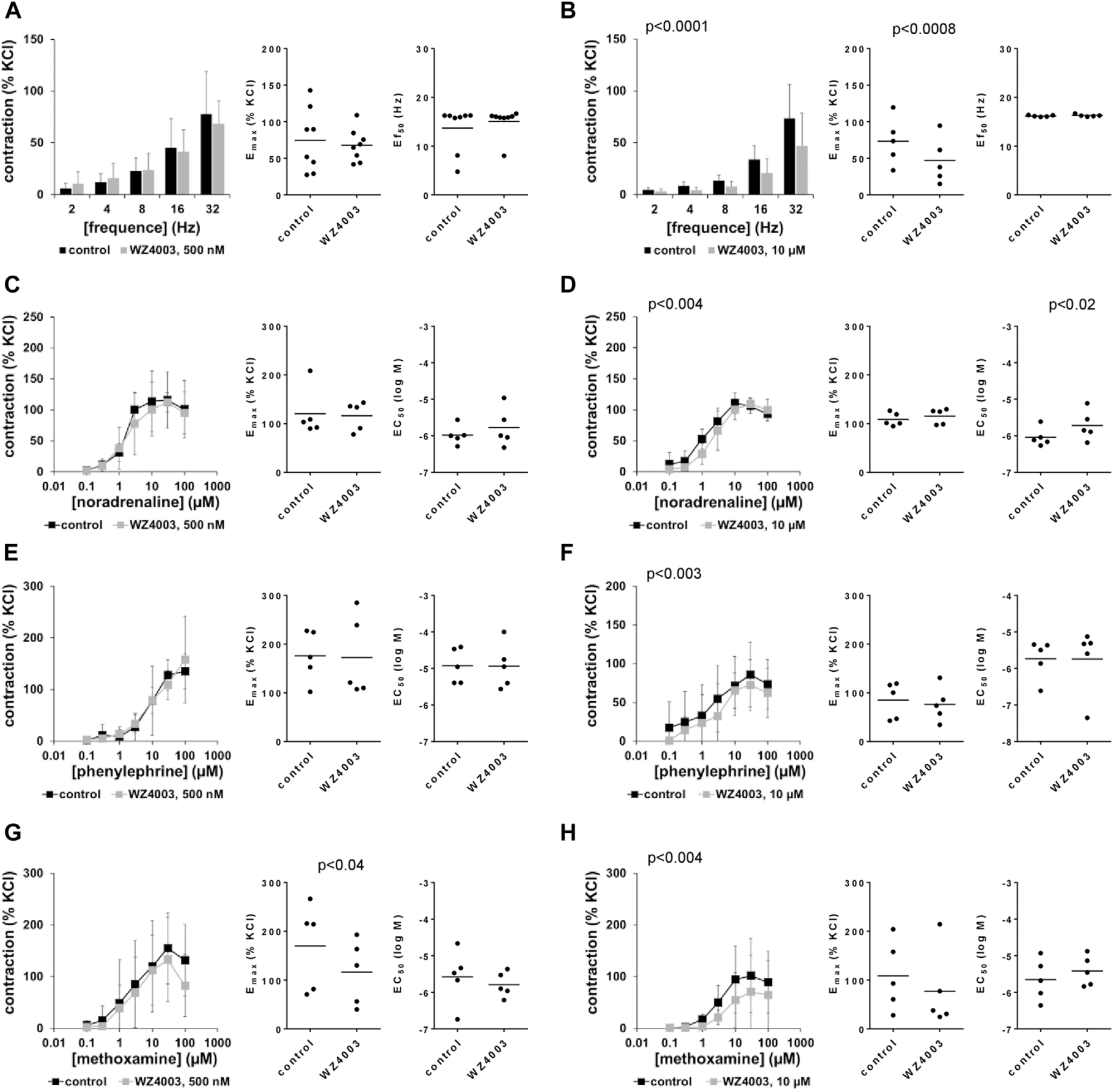
Effects WZ4003 on EFS-induced and adrenergic contractions of human prostate tissues. Contractions of human prostate tissues were induced by EFS **(A,B)**, noradrenaline **(C,D)**, phenylephrine **(E,F)** or methoxamine **(G,H)** in an organ bath, 30 min after addition of WZ4003 in concentrations of 500 nM **(A,C,E,G)** or 10 µM **(B,D,F,H)** or of an equivalent amount of DMSO (controls), which was used as solvent for WZ4003. Shown are data from *n* = 8 independent experiments in **(A)** and *n* = 5 independent experiments in each other subpanel, where tissues from *n* = 8 or *n* = 5 patients were used for both groups of a subpanel, resulting in paired samples. Shown are means ± SD from all experiments in concentration response curves with *p* values from two-way ANOVA for whole groups, and all single E_max_, EC_50_ and Ef_50_ values from all experiments (calculated by curve fitting) together with *p* values from Student’s *t*-test in scatter plots.

EFS-induced contractions were decreased by 10 μM, but not by 500 nM WZ4003 (Figures 10A, B). Decreases by 10 µM WZ4003 amounted to 41% (22–61) at 32 Hz, and were reflected by decreases of E_max_ values by 41% (22–61) (Figure 10B). Ef_50_ values were not changed by WZ4003 (Figures 10A, B).

### 3.15 Effects of WZ4003 on adrenergic contractions of human prostate tissues

Contractions by α_1_-adrenergic agonists were not affected by WZ4003 (Figures 10C–F), apart from a decrease in E_max_ for methoxamine by 32% (12–52) by 500 nM WZ4003 (attributed to a decreased contraction at 100 µM methoxamine) (Figure 10G), and from slight decreases of contractions seen concentration response curves for methoxamine (Figure 10H).

### 3.16 Effects of WZ4003 on non-adrenergic contractions of human prostate tissues

**FIGURE 11:**
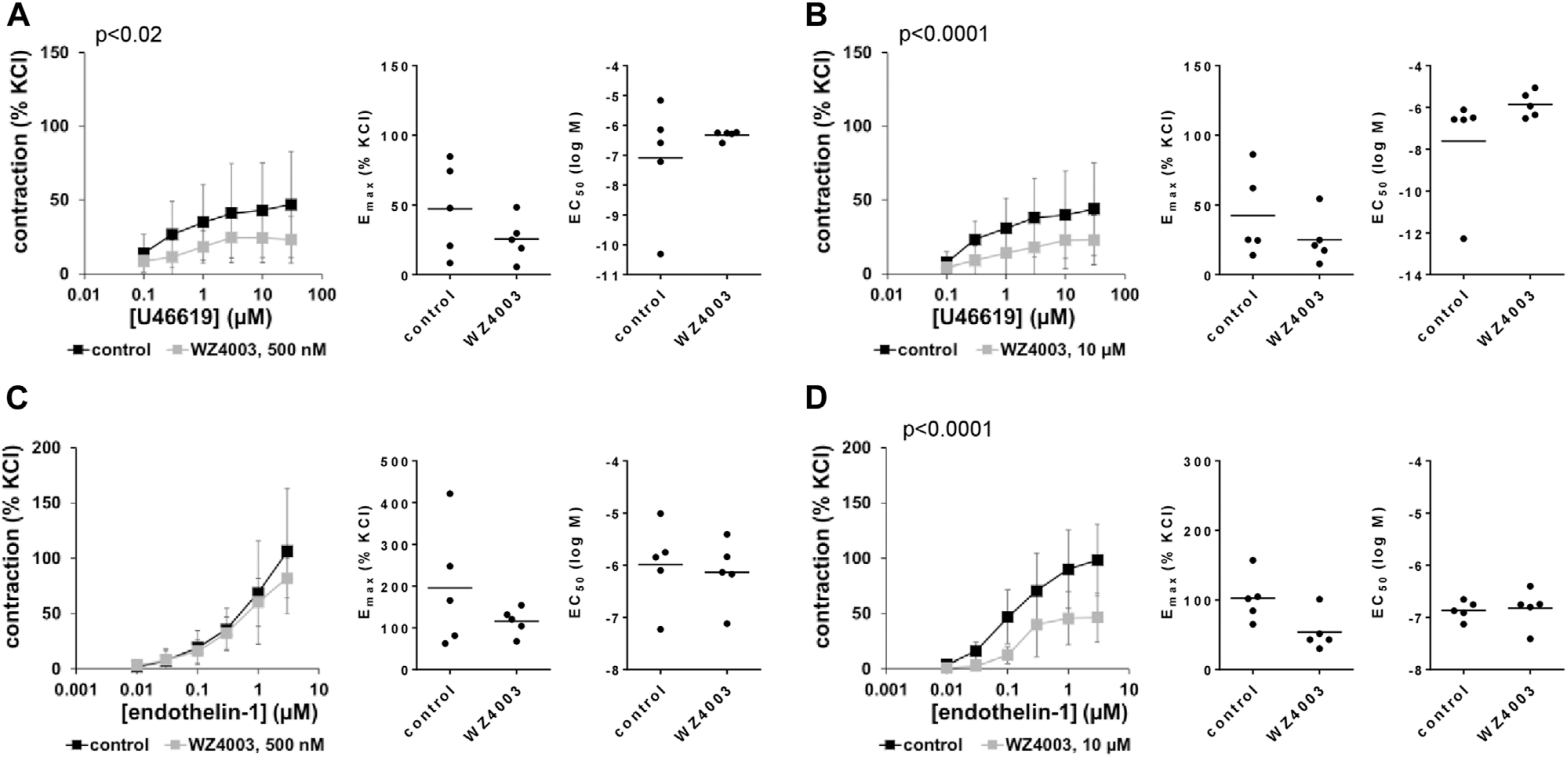
Effects of WZ4003 on non-adrenergic contractions of human prostate tissues. Contractions of human prostate tissues were induced by U46619 **(A,B)** or endothelin-1 **(C,D)** in an organ bath, 30 min after addition of WZ4003 in concentrations of 500 nM **(A,C)** or 10 µM **(B,D)** or of an equivalent amount of DMSO (controls), which was used as solvent for WZ4003. Shown are data from *n* = 5 independent experiments in each subpanel, where tissues from *n* = 5 patients were used for both groups of a subpanel, resulting in paired samples. Shown are means ± SD from all experiments in concentration response curves with *p* values from two-way ANOVA for whole groups, and all single E_max_ and EC_50_ values from all experiments (calculated by curve fitting) in scatter plots.

U46619-induced contractions were inhibited by 500 and 10 µM WZ4003, to obviously similar degree in concentration response curves (Figures 11A, B). Decreases seen in concentration response curves were reflected by decreases in E_max_ values, amounting to 16% (−91–123) [54% (19–88), if one outlier was excluded] with 500 nM HTH01-015, and to 37% (17–58) with 10 µM HTH01-015 (Figures 11A, B). EC_50_ values for U46619 were increased by more than half a magnitude (MD (logM) 0.7 (−1.5–3.0) by 500 nM WZ4003, and by 1.7 magnitudes (−1.6–5.1) by 10 µM WZ4003 (Figures 11A, B).

Endothelin-1-induced contractions were inhibited by 10 μM, but not by 500 nM WZ4003 (Figures 11C, D). Decreases seen by 10 µM in concentration response curves were reflected by decreases in E_max_ values, amounting to 43% (6–80) (Figure 11D). EC_50_ values for endothelin-1 were not affected by WZ4003 (Figures 11C, D).

## 4 Discussion

Our findings point to NUAK-mediated suppression of cell death and NUAK-mediated proliferation in prostate stromal cells, while NUAK-driven smooth muscle contraction in the prostate appears limited. Silencing experiments confirmed similar functions of NUAK1 and NUAK2 in cultured stromal cells, which were mimicked by the presumed NUAK inhibitors HTH01-015 and WZ4003. Complementary to our findings in WPMY-1 cells, both inhibitors shared similar, although limited anticontractile effects in contraction experiments with human prostate tissues, which were most obvious for neurogenic and thromboxane-induced contractions. Growth-related functions, including proliferation and cell death, alone or together with smooth muscle contraction are critical in etiology and medical treatment of voiding symptoms suggestive of BPH, where hyperplastic growth and elevated smooth muscle tone in the prostate may cause urethral obstruction, resulting in symptoms by impaired urinary flow.

NUAK1 and -2 were both detectable in WPMY-1 cells, confirming their expression in prostate stromal cells. In human prostate tissues, glandular epithelial cells and catecholaminergic neurons may express NUAK1 and -2 as well, as mRNA levels correlated positively with markers for these cell types. NUAK1 expression showed a binominal distribution in human prostate tissues, with expression levels in six samples clearly exceeding the levels in 14 other examined samples. High NUAK1 mRNA levels in these samples may be imparted by infiltrating cells, as they did not correlate positively with any marker for prostate cells. An involvement of chronic inflammation is increasingly considered in pathogenesis of BPH; approximately 40% of biopsy specimens from patients with BPH contain inflammatory infiltrates (De Nunzio et al., 2016). Positive correlation of NUAK2 with PSA in prostate tissues suggests that expression of this isoform increases with BPH or, more specifically, with glandular hyperplasia, considering the correlation with keratin-19. In fact, BPH may be imparted by stromal, epithelial or mixed hyperplasia, which may both contribute to voiding symptoms (Strand et al., 2017). However, findings at mRNA levels can be of limited conclusiveness, compared to protein and functional levels. In fact, our Western blot analyses did not point to correlations of NUAK expression with cytokeratins, but the number of examined tissues was low.

The impact of silencing was highest on cell death, proliferation and actin polymerization, and still obvious on viability. Silencing of either isoform substantially increased the number of dead cells and reduced the proliferation of WPMY-1 cells. Our findings are in line with previous studies reporting increases in cell death by NUAK1 silencing in colorectal cancer cells (Suzuki et al., 2004), reduced survival of breast cancer cells by silencing of NUAK2 (Kim et al., 2008), and reduced proliferation of cervical cancer cells by silencing of NUAK2 (Li et al., 2021). Effects of silencing on cell death, proliferation and viability in our study were mimicked by HTH01-015 and WZ4003. Inhibitor effects were largest using 10 μM, but lacking or small using 2.5 µM. Exceptions were the proliferation rate and actin organization, the first still being reduced by 2.5 µM WZ4003 and the latter by 2.5 µM of both inhibitors, while lower concentrations affected both functions only to smallest extent. Decreased expression of the smooth muscle marker calponin and of Ki-67 seen after exposure of prostate tissues with 10 µM HTH01-015 or WZ4003 may reflect a net effect from cell death and reduced proliferation, confirming our findings from cultured stromal cells in tissues. Whether missing effects on cytokeratin expression in tissues truly reflect lacking regulation of survival of glandular epithelial cells by NUAK1 or -2, requires further examination by separate studies. The potency and efficacy of both inhibitors differed with cell types in previous studies. In U2OS cells, 3 µM of HTH01-015 or WZ4003 virtually abolished mitosis (Banerjee et al., 2014b), while 10 µM reduced proliferation by half in U2OS cells but completely in mouse embryonic fibroblasts (Banerjee et al., 2014a). In another study, 10 µM WZ4003 obviously increased apoptosis in HCT116 cells, but not or to neglectable extent in U2OS and SW480 cells, whereas 2 µM nearly abolished colony formation in HCT116 and SW480 but not in U2OS cells, and 10 µM but not 2.5 µM reduced proliferation in all 3 cell types (Yang et al., 2021).

In biochemical assays with recombinant kinases, WZ4003 inhibited NUAK1 with an IC_50_ of 20 nM and NUAK2 with an IC_50_ of 100 nM, whereas HTH01-015 inhibited only NUAK1 with an IC_50_ of 100 nM, but not NUAK2 (Banerjee et al., 2014a). The specificity of both inhibitors was addressed by a screening assay including 139 kinases (Banerjee et al., 2014a). In these assays, NUAK1 was inhibited by 89% by 1 µM HTH01-015, and by 94% by 1 µM WZ4003 (Banerjee et al., 2014a). However, the specificity may be limited, as 1 µM WZ4003 inhibited eleven other kinases by >40% in these assays, including STK33 and IGF-1R (both inhibited by 67%), ULK2 and ULK1 (inhibited by 63% or 48%, respectively), CAMKKβ (56%), MARK3 (49%), TTK (46%), PKD1 (43%), CHK1 and CHK2 (inhibited by 42% and 60%) and TSSK1 (42%), while 30 further kinases were inhibited between 21% and 37% and activities of all other 98 kinases ranged between 80% and 120% of controls (i.e., ≤20% inhibition) (Banerjee et al., 2014a). HTH01-015 inhibited only one other kinase by more than 40% (CLK2, 46%) and four other kinases between 24% and 32%, while activities of all other 134 kinases ranged between 81% and 133% of controls (i.e., ≤19% inhibition) (Banerjee et al., 2014a). Off-targets included MLC kinase, which is critical in smooth muscle contraction and was inhibited by 23% by 1 µM HTH01-015 (Banerjee et al., 2014a). While an even larger inhibition may be expected in our experiments with 10 μM, 10 µM did not affect MLC phosphorylation in our incubation experiments with prostate tissues, suggesting that MLC kinase inhibition did not become relevant in our tissues. Together, off-target effects appear possible and may (but must not necessarily) contribute to effects seen with micromolar inhibitor concentrations, but may be small in our experiments with 500 nM. As another limitation, our study did not include experiments with overexpression of NUAKs, or with constructs expressing NUAKs with loss of function mutations.

Notably, our study is to the best of our knowledge the first addressing effects of NUAK silencing and of NUAK inhibitors on smooth muscle contraction, despite previous evidence supporting the idea of NUAK-mediated contraction (Sun et al., 2013; Faisal et al., 2021). In general, contraction of any smooth muscle type requires MLC phosphorylation, polymerization of actin, and its organization to filaments (Hennenberg et al., 2014). MLC phosphorylation is critically regulated by MLC phosphatase, consisting of different subunits, including the myosin-targeting subunit MYPT1, which is in turn regulated by phosphorylation at different sites. Silencing of NUAK1 inhibited MLC phosphorylation and contraction in non-muscle cells, which was obviously imparted by MYPT1 regulation (Zagorska et al., 2010). Similarly and again in non-muscle cells, silencing of NUAK2 reduced MLC phosphorylation (Vallenius et al., 2011). Overexpression or silencing of NUAK1 and -2 in non-muscle cells demonstrated promotion of actin polymerization and filament organization by both isoforms (Zagorska et al., 2010; Vallenius et al., 2011). Consequently, NUAK1 silencing inhibited actin-dependent cell functions, including migration and adhesion (Zagorska et al., 2010; Banerjee et al., 2014a). Inhibition of MYPT1 phosphorylation was previously observed with HTH01-015 and WZ4003, but required concentrations as high as 10 or 3 µM (Banerjee et al., 2014a). These previous findings supported the idea that NUAKs may drive smooth muscle contraction (Sun et al., 2013; Faisal et al., 2021), and prompted us to examine the impact of NUAK silencing on contractions of WPMY-1 cells, and of NUAK inhibitors on human prostate smooth muscle contraction.

Our findings from cell culture and tissues suggest, that NUAK functions in prostate smooth muscle contraction are limited. Even though silencing of NUAK1 or NUAK2 resulted in reduced contractions, this inhibition was partial and may be explained by cell death or by inhibition of proliferation as well, in addition to a NUAK-driven mechanism of smooth muscle contraction. In our organ bath experiments, cell death as a reason underlying anticontractile effects appears unlikely, as this should affect all agonist-induced contractions, and because inhibitors were applied for only 30 min, whereas incubation with 10 µM for 5 h did not affect the content of the smooth muscle marker calponin. Using 500 nM in organ bath experiments with human prostate tissues, we observed partial inhibitions of EFS-induced contractions by HTH01-015, and of U46619-induced contractions by both inhibitors, while α_1_-adrenergic and endothelin-1-induced contractions remained unaffected. Although we can not explain the differential effects on EFS, or why effects on agonist-induced contractions were selective for U46619, we suppose that at least the inhibition of EFS- and U46619-induced contractions were truly imparted by NUAK inhibition, due to the concentration in a nanomolar range together with reported IC_50_ values and inhibition data for other kinases (Banerjee et al., 2014a). Using concentrations of 10 μM, inhibition of endothelin-1-induced contractions by both inhibitors and inhibition of α_1_-adrenergic contractions by HTH01-015 added to effects seen by 500 nM.

Similarly, we suppose that none of the effects seen in organ bath experiments with 500 nM was imparted by reduced MLC phosphorylation, first because HTH01-015 did not affect MLC phosphorylation in human prostate tissues, and secondly as reduced MLC phosphorylation should affect all agonist-induced contractions, but not only the U46619-induced. NUAK1 phosphorylates MYPT1 at serines 472 and 445 (Zagorska et al., 2010), and is the only known kinase phosphorylating serine-472, while phosphorylation at serine-445 is shared with LATS1/WARTS (Chiyoda et al., 2012). Reduced serine-472 phoshophorylation may confirm NUAK inhibition by 500 nM HTH01-015 in our experiments with tissues, and suggested phosphorylation of this position by both isoforms in our silencing experiments. In contrast to NUAK1-mediated phosphorylation, and other than previously assumed (Banerjee et al., 2014a), previous evidence proving NUAK2-mediated MYPT1 phosphorylation at serine-472, e.g., from silencing or transgenic models is to the best of our knowledge not available. Although phosphorylation at sites other than threonine 696 and 853 has been reported for NUAK2, identification of precise positions was not yet possible (Yamamoto et al., 2008).

Serine-445- and -472 phosphorylations were reduced by silencing in WPMY-1 cells and by HTH01-015 in prostate tissues, whereas MLC phosphorylation was regulated asynchronously, being reduced by silencing in cell culture experiments, but unchanged by HTH01-015 in tissues. Apart from NUAK-mediated phosphorylation, MLC phosphatase regulation underlies regulation by Rho kinase, which inhibits MLC phosphatase by MYPT1 phosphorylation at threonine-696. We assume that the asynchronous MLC regulation in cells and tissues was imparted by divergent MYPT1 phosphorylation at threonine-696, which obviously differed between cells and tissues. In fact, threonine-696-phosphorylated MYPT1 was detectable in tissues, but undetectable in cultured WPMY-1 cells. Consequently, it appears possible that the MLC phosphatase activity is lower in tissues compared to cultured cells and thus, levels of basal MLC phosphorylation are higher in tissues compared to WPMY-1 cells, together preventing (detectable) changes in MLC phosphorylation by NUAK inhibition in tissues. Thus, a high constitutive threonine-696 phosphorylation in tissues (lacking under cell culture conditions) may cover changes in MLC phosphatase activity by NUAK inhibition in tissues. As threonine-696 phosphorylation is mediated by contractile agonists, we speculate that the high phosphorylation in tissues may originate from paracrine or neuronal mediators, which may still be released *ex vivo*. Candidates are thromboxane A_2_, supposed to be produced by epithelial cells and acting on stromal cells (Hennenberg et al., 2014), or noradrenaline, released from residual neurons, both lacking in cell culture. Threonine-696 phosphorylation of MYPT1 by NUAK2 has been previously suggested as well (Lessard et al., 2016), but was not affected by 500 nM HTH01-015 in our experiments with prostate tissues. Finally, the precise contributions from NUAK-mediated MYPT1 regulation to total MLC phosphatase activity are unknown, and relationships between different phosphorylations and their contributions to net activity are still elusive. Thus, the divergent MLC regulation seen in cultured cells and tissues may reflect differences between both models, and may point to a higher level of complexitiy in MLC phosphatase regulation *in situ* or *in vivo*, compared to cultured cells.

A role of NUAKs in MLC phosphorylation has been repeatedly proposed, but available evidence is surprisingly limited. Previous data showing inhibition of MLC phosphorylation by NUAK inhibitors are obviously not available, even though inhibition of serine-445 phosphorylation at MYPT1 by HTH01-015 has been shown (Banerjee et al., 2014a). Another study reported reduced MYPT1 phosphorylation by knockout of NUAK1 in mouse embryonic fibroblasts and HEK293 cells, whereas MLC phosphorylation in knockouts was not reduced under basal conditions, but only under certain conditions in complex biochemical assays (Zagorska et al., 2010). Silencing of NUAK2, however, in fact reduced MLC phosphorylation in HeLa cells (Vallenius et al., 2011). Together, our findings are in line with previous data, but point to a complex regulation of MYPT1, including but not solely depending on NUAK-mediated MYPT1 regulation, and including contributions lacking under cell culture conditions but critically influencing MLC phosphatase activity in tissues or *in vivo*.

At least in experiments using inhibitor concentrations of 500 nM, the breakdown of actin organization seen with HTH01-015 and WZ4003 was possibly caused by NUAK inhibition, rather than off-target effects. However, this breakdown can not explain anticontractile effects in our organ experiments, as this should affect any, but not only EFS- or U46619-induced contractions. Although we observed actin depolymerization by silencing and NUAK inhibitors in WPMY-1 cells, this was examined 48 h after silencing, or 24 h after inhibitor application, whereas contractions in tissues were induced 30 min after inhibitor application. Together, mechanisms underlying inhibition of contractions by both inhibitors and by silencing remain elusive at this stage and require further studies.

Voiding symptoms in BPH, characterized by weak urinary flow and impaired bladder emptying, are caused by urethral obstruction due to prostate enlargement and increased prostate smooth muscle tone (Lepor, 2004; Chapple, 2011). Increased prostate size can be imparted by stromal and glandular-epithelial hyperplasia, occuring alone or together (Strand et al., 2017). Our study focussed on stromal cells, so that an equivalent function for growth of glandular epithelial cells remains pending. Considering that effects of NUAK silencing on cell death and proliferation of WPMY-1 cells were of quite large extent, a critical role of both isoforms may be supposed for prostate growth in BPH. Medical treatment in BPH aims to reduce prostate size by inhibition of prostate growth, and to reduce prostate smooth muscle tone (Lepor, 2004; Oelke et al., 2013). Available drugs include 5α-reductase inhibitors to reduce prostate volume, and α_1_-adrenoceptor antagonists and the phosphodiesterase-5 inhibitor tadalafil supposed to inhibit prostate smooth muscle contraction (Oelke et al., 2013). Even though medical treatment is commonly prescribed, limitations are obvious, and the number of surgeries for BPH is still high (Magistro and Stief, 2020). α_1_-Blockers are the first line option, and reduce international prostate symptom scores (IPSS) by 30%–50%, and improve the maximum urinary flow rate (Q_max_) by 20%–40% in uncontrolled studies with placebo run-in (Oelke et al., 2013). In open-label studies without run-in phase, decreases in IPSS up to 50% and increases in Q_max_ up to 40% are observed (Oelke et al., 2013). Placebo effects are large (Eredics et al., 2017), approaching to decreases in IPSS of 30% or more, and to improvements of Q_max_ of at least 15% (Kortmann et al., 2003; Madersbacher et al., 2007; Chapple et al., 2011; Oelke et al., 2013). The number of non-responders, i.e., patients experiencing decreases in IPSS by not more than 25% amounts to 30%–35%, so that 69% of patients are disappointed from α_1_-blockers, and 65% discontinue the medication within 1 year after first prescription (Chapple et al., 2011; Fullhase et al., 2013; Matsukawa et al., 2013; Cindolo et al., 2015a; Cindolo et al., 2015b; Lee et al., 2015). Tadalafil improves IPSS to similar extent as α_1_-blockers, but does not increase Q_max_ (Pattanaik et al., 2018). 5α-Reductase inhibitors are applied to prevent progression, complications and surgery, but reduce prostate size by not more than 28%, and improvements of symptoms or urinary flow are not additive with α_1_-blockers in combined treatment (Oelke et al., 2013).

In the face of these limitations, together with increasing case numbers due to demographic transitions and the age-dependencies of prevalence and natural history, novel medications are of high demand. New drugs can only emerge from adequate, improved understanding of molecular mechanisms underlying growth and smooth muscle contraction in the prostate, and from identification of novel targets. Limited efficacy of α_1_-blockers has been attributed to non-adrenergic contractions, which are insensitive to α_1_-blockers and may keep prostate smooth muscle tone elevated during medical treatment (Hennenberg et al., 2014; Hennenberg et al., 2017). Whether inhibition of thromboxane-induced contractions by NUAK inhibitors, observed in our study, is sufficient to translate to a benefit or to urodynamic effects *in vivo*, remains uncertain at this stage. Addressing the effects of NUAK inhibitors on experimentally-induced symptoms, or on prostate size in animal models of BPH merits further attention.

## 5 Conclusion

NUAK1 and -2 suppress cell death and promote proliferation in prostate stromal cells. Effects of NUAK silencing are mimicked by HTH01-015 and WZ4003, which requires, at least partly, high inhibitor concentrations. While previous studies reporting MLC phosphorylation by NUAKs suggested a possible role of NUAKs in driving smooth muscle contraction, a procontractile role in stromal cells and in human prostate smooth muscle appears limited for both NUAK isoforms. Considering large effects of NUAK silencing on growth-related functions in stromal cells, a role in stromal hyperplasia in BPH and effects of NUAK inhibitors on prostate growth appear possible.

## Data Availability

The original contributions presented in the study are included in the article/Supplementary Material, further inquiries can be directed to the corresponding author.
